# Analysis of the cartilage proteome from three different mouse models of genetic skeletal diseases reveals common and discrete disease signatures

**DOI:** 10.1242/bio.20135280

**Published:** 2013-06-18

**Authors:** Peter A. Bell, Raimund Wagener, Frank Zaucke, Manuel Koch, Julian Selley, Stacey Warwood, David Knight, Raymond P. Boot-Handford, David J. Thornton, Michael D. Briggs

**Affiliations:** 1 Wellcome Trust Centre for Cell–Matrix Research, Faculty of Life Sciences, The University of Manchester, Manchester M13 9PT, UK; 2 Center for Biochemistry, University of Cologne, D50931 Cologne, Germany; 3 Center for Molecular Medicine, University of Cologne, D50931 Cologne, Germany; 4 Institute for Dental Research and Musculoskeletal Biology, Medical Faculty, University of Cologne, D50931 Cologne, Germany; * Present address: Institute of Genetic Medicine, Newcastle University, International Centre for Life, Newcastle upon Tyne NE1 3BZ, UK

**Keywords:** Cartilage, Genetic skeletal disease, Proteomics, Pseudoachondroplasia, Multiple epiphyseal dysplasia

## Abstract

Pseudoachondroplasia and multiple epiphyseal dysplasia are genetic skeletal diseases resulting from mutations in cartilage structural proteins. Electron microscopy and immunohistochemistry previously showed that the appearance of the cartilage extracellular matrix (ECM) in targeted mouse models of these diseases is disrupted; however, the precise changes in ECM organization and the pathological consequences remain unknown. Our aim was to determine the effects of matrilin-3 and COMP mutations on the composition and extractability of ECM components to inform how these detrimental changes might influence cartilage organization and degeneration.

Cartilage was sequentially extracted using increasing denaturants and the extraction profiles of specific proteins determined using SDS-PAGE/Western blotting. Furthermore, the relative composition of protein pools was determined using mass spectrometry for a non-biased semi-quantitative analysis.

Western blotting revealed changes in the extraction of matrilins, COMP and collagen IX in mutant cartilage. Mass spectrometry confirmed quantitative changes in the extraction of structural and non-structural ECM proteins, including proteins with roles in cellular processes such as protein folding and trafficking. In particular, genotype-specific differences in the extraction of collagens XII and XIV and tenascins C and X were identified; interestingly, increased expression of several of these genes has recently been implicated in susceptibility and/or progression of murine osteoarthritis.

We demonstrated that mutation of matrilin-3 and COMP caused changes in the extractability of other cartilage proteins and that proteomic analyses of *Matn3* V194D, *Comp* T585M and *Comp* DelD469 mouse models revealed both common and discrete disease signatures that provide novel insight into skeletal disease mechanisms and cartilage degradation.

## Introduction

The chondrodysplasias are a diverse group of many different phenotypes that arise when endochondral bone growth is disrupted and include two clinically related phenotypes; multiple epiphyseal dysplasia (MED) and pseudoachondroplasia (PSACH) (Warman et al., 2011). MED is genetically heterogeneous and can be caused by mutations in genes encoding the extracellular matrix (ECM) proteins matrilin-3, type IX collagen and cartilage oligomeric matrix protein (COMP) (Briggs and Chapman, 2002; Jackson et al., 2012); PSACH results exclusively from *COMP* mutations (Jackson et al., 2012) and is more severe than MED, but both phenotypes comprise a disease spectrum with symptoms that can include joint pain and stiffness, lower-limb deformities and early onset osteoarthritis (Briggs and Chapman, 2002).

We have previously generated targeted mouse models of PSACH-MED with mutations in matrilin-3 (moderate MED: *Matn3* V194D) and COMP (mild PSACH: *Comp* T585M and severe PSACH: *Comp* DelD469) and described the resulting phenotypes in detail (Leighton et al., 2007; Piróg-Garcia et al., 2007; Suleman et al., 2012). Briefly, all three mice models exhibit disproportionate short stature due to decreased chondrocyte proliferation and increased and/or spatially dysregulated apoptosis in the cartilage growth plate.

Whilst the cellular response to mutant matrilin-3 and COMP expression has been documented in detail (Bell et al., 2012; Leighton et al., 2007; Piróg-Garcia et al., 2007; Suleman et al., 2012), the effects of these mutant proteins on the organisation and composition of the cartilage ECM are not clearly defined. Immunohistochemical (IHC) analysis has previously identified differences in the staining pattern for matrilin-3, COMP and type IX collagen in the ECM of mutant growth plates (Leighton et al., 2007; Piróg-Garcia et al., 2007; Suleman et al., 2012), whilst the appearance of the ECM ultra-structure is also different in all three mutant mice. For example, the collagen fibrils in the inter-territorial matrix were more clearly visible by electron microscopy (EM) suggesting that lower levels of fibril surface-associated proteins were decorating individual collagen fibrils (Leighton et al., 2007; Piróg-Garcia et al., 2007; Suleman et al., 2012). Similar changes to the organisation of the cartilage pericellular matrix in mice expressing a *COMP* DelD469 transgene were also noted by EM and IHC (Schmitz et al., 2008). Furthermore, recent studies demonstrated that a secreted variant of COMP carrying a MED-mutation in the C-terminal domain (p.H587R) disrupted collagen fibrillogenesis *in vitro* and also in a cell culture model (Hansen et al., 2011). In contrast, COMP-null mice show no differences in collagen fibril diameter (Svensson et al., 2002) and these data therefore suggest that PSACH and MED-causing mutations in matrilin-3 and COMP can cause changes in the ultra-structure of the cartilage ECM through antimorphic mechanisms. However, the extent to which these changes compromise the organisation and function of the ECM, and therefore contribute to disease pathology and cartilage degradation, remain to be determined.

The unique biochemical properties of cartilage have made the study of its ECM technically difficult relative to other tissues (Wilson et al., 2009). Recently, however, methods have been described that allow the reproducible analysis of cartilage proteins by Western blotting, 2-D gel electrophoresis and mass spectrometry (MS) (Wilson et al., 2009). However, these methods have not been employed to study cartilage preparations from a heterogeneous series of gene-targeted mouse models of genetic skeletal diseases.

We hypothesised that the expression of mutant matrilin-3 and COMP would induce changes to the extractability of other cartilage components, which would be indicative of alterations to the anchoring or associations of individual molecules and the overall functional properties of the tissue. We tested this hypothesis by analysing the proteins isolated from sequential extractions of cartilage from mutant mouse models with different genotypes. This study describes for the first time the proteomic characterisation of cartilage from *Matn3* V194D, *Comp* T585M and *Comp* DelD469 mutant mice using a combination of Western blotting and semi-quantitative spectral counting (mass spectrometry) and identifies both common and unique disease signatures that also provide insight into detrimental changes that may predispose to cartilage degeneration.

## Results

### A candidate approach reveals the effects of matrilin-3 and COMP mutations on cartilage protein extractability

**Fig. 1.**
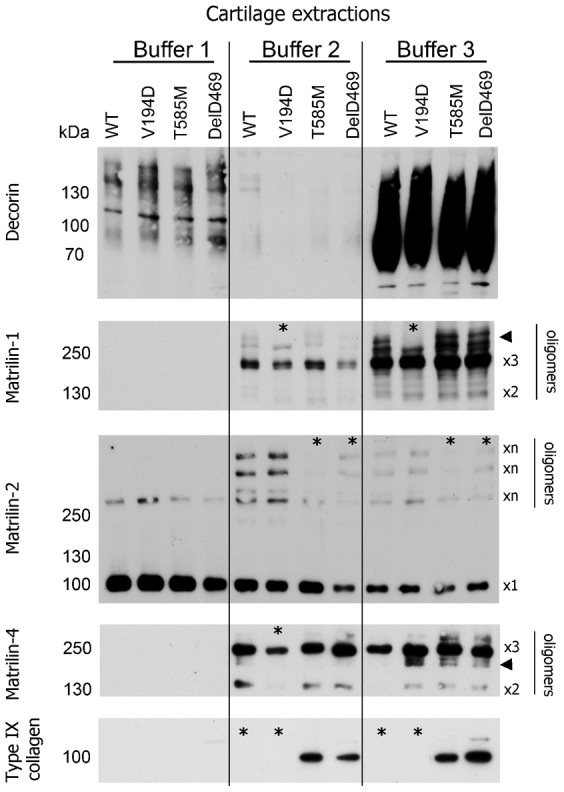
SDS-PAGE and Western blotting show genotype differences in the extractability of specific cartilage structural proteins. Cartilage from wild type (WT) and mutant (*Matn3* V194D, *Comp* T585M and *Comp* DelD469) mice was extracted in a series of three buffers (Buffer 1, 2 and 3). Proteins were separated by SDS-PAGE under non-reducing conditions and analyzed by Western blotting using antibodies specific to decorin, matrilin-1, -2 and -4 and type IX collagen. Higher-order oligomeric forms of matrilin-1 and smaller oligomeric forms of matrilin-4 are indicated by arrowheads. Key: ×1 = putative monomers; other putative oligomeric forms of proteins are also indicated (×2 = dimers, ×3 = trimers, ×n = undetermined molecular forms); * denotes differences detected in protein extraction profiles between mice of different genotypes; kDa = mass in kilo Daltons.

**Fig. 2.**
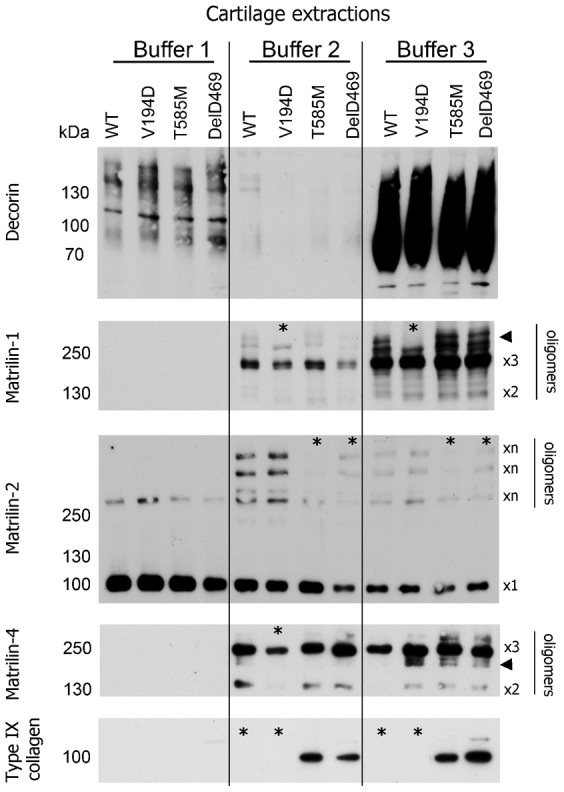
SDS-PAGE and Western blotting show genotype differences in the extractability of matrilin-3 and COMP. Cartilage from wild type (WT) and mutant (*Matn3* V194D, *Comp* T585M and *Comp* DelD469) mice was extracted in a series of three buffers (Buffer 1, 2 and 3). Proteins were separated by SDS-PAGE under non-reducing conditions (unless stated otherwise) and analyzed by Western blotting using antibodies specific to (**A**) matrilin-3 and (**B**) COMP. The boxed area is shown at higher magnification in the bottom panel of A. Key: ×1 = putative monomers; other putative oligomeric forms of proteins (×2 = dimers, ×3 = trimers, ×4 = tetramers); * denotes differences detected in protein extraction profiles between mice of different genotypes; kDa = mass in kilo Daltons.

In the first instance we used a candidate approach to determine the extractability of a number of ECM molecules including members of the matrilin protein family, type IX collagen and decorin, which are all known to interact with matrilin-3 and COMP (Budde et al., 2005; Fresquet et al., 2010; Fresquet et al., 2007; Holden et al., 2001; Mann et al., 2004; Wiberg et al., 2003). Articular and epiphyseal cartilage from 3-week-old mouse knee joints was sequentially extracted in a series of three buffers and the extracted proteins were separated by SDS-PAGE and visualised by Western blotting. The extraction of decorin appeared consistent between all genotypes and was present primarily in buffer 3 (Fig. 1); in contrast, the extraction of matrilins 1–4, type IX collagen and COMP all showed obvious genotype-specific differences in one or more of the disease models (Figs 1, 2). Whilst there were numerous minor changes in protein extractability, particularly slight differences in oligomeric forms, we chose to concentrate on the more obvious changes, which we considered would have the greatest influence on ECM organization.

Matrilin-1 was slightly less extractable from *Matn3* V194D and *Comp* DelD469 cartilage with buffer 2 and several higher-order oligomeric forms of matrilin-1 were not extractable from *Matn3* V194D cartilage, but were readily extracted from the cartilage of all other genotypes (Fig. 1). There was also a reduction in the amount of matrilin-2 oligomers extracted in buffers 2 and 3 from *Comp* T585M and *Comp* DelD469 cartilage relative to the control and *Matn3* V194D cartilages (Fig. 1). A decrease in the extraction of matrilin-4 dimers and trimers from *Matn3* V194D cartilage buffer 2 samples was observed compared to the other genotypes, and an increase in the extraction of some matrilin-4 oligomers that were slightly smaller in size than full-length trimers was also noted in all three mutant genotypes relative to the wild type control (Fig. 1).

The most striking difference was that it was not possible to extract type IX collagen from either wild type or *Matn3* V194D cartilage using any of the buffer conditions, whereas a single molecular form corresponding in size to full length α1(IX) chain was readily extractable from both *Comp* DelD469 and T585M cartilages using buffers 2 and 3 (Fig. 1). Significantly, these changes in the extractability of type IX collagen were not the result of differences in the total levels of this protein since IHC and microarray analyses have consistently demonstrated comparable levels in mice of all three mutant genotypes that are similar to wild type controls (supplementary material Figs S1, S2) (Leighton et al., 2007; Piróg-Garcia et al., 2007; Suleman et al., 2012).

### Extractability of matrilin-3 from cartilage is increased in *Comp* DelD469 and *Comp* T585M mice compared with wild type controls

In agreement with previous studies showing the intracellular aggregation of mutant matrilin-3 (Bell et al., 2012), we observed that matrilin-3 was extracted with buffers 1–3 from *Matn3* V194D cartilage as a high molecular weight aggregate under non-reducing conditions (Fig. 2A). We also noted that a small amount of matrilin-3 was also extracted with buffer 1 in *Comp* DelD469 and *Comp* T585M cartilages compared to wild type (Fig. 2A, insert). Furthermore, there were also differences in the molecular forms of matrilin-3 that were extracted from *Comp* DelD469 and *Comp* T585M cartilages. In *Comp* T585M cartilage two oligomeric forms of approximately 200 kDa were extracted in near equal quantities, whilst the smaller of these two forms was entirely absent in *Comp* DelD469 cartilage (Fig. 2A, insert). The sizes of these oligomers suggested that they may represent both intact and a smaller proteolytically processed matrilin-3 tetramer. There were no discrete oligomers present in *Matn3* V194D cartilage.

### Extractability of COMP is increased in *Matn3* V194D, *Comp* DelD469 and *Comp* T585M mice compared with controls

Analysis of cartilage sequential extractions revealed differences in the extractability of COMP between mice of all three genotypes (Fig. 2B). For example, when analyzed under non-reducing conditions a greater quantity of COMP was extracted in buffers 1 and 2 from *Matn3* V194D cartilage compared to both *Comp* mutant mice and the wild type control (Fig. 2B, top panel). The extraction of COMP from *Comp* T585M and *Comp* DelD469 mice was also increased in all buffers compared to the wild type control, although the majority of COMP from both *Comp* T585M and *Comp* DelD469 cartilage was extracted with buffer 3, which was in contrast to *Matn3* V194D cartilage. Finally, COMP extracted from all mutant genotypes appeared larger in apparent molecular weight compared to COMP extracted from wild type cartilage under non-reducing conditions.

Interestingly, when protein samples were resolved under reducing conditions Western blotting consistently showed the complete absence of a single COMP fragment (∼90 kDa) from both *Matn3* V194D and *Comp* DelD469 buffer 2 and buffer 3 extractions, which might represent full-length monomeric COMP (Fig. 2B, bottom panel). Surprisingly, a number of higher migrating bands were also detected by the COMP antibody (between ∼120–150 kDa) in all three genotypes and these proteins were most prevalent in buffer 3 samples; however, the nature of these bands remains unclear.

### Wild type matrilin-3 is co-retained with mutant COMP in *Comp* DelD469 chondrocytes

**Fig. 3.**
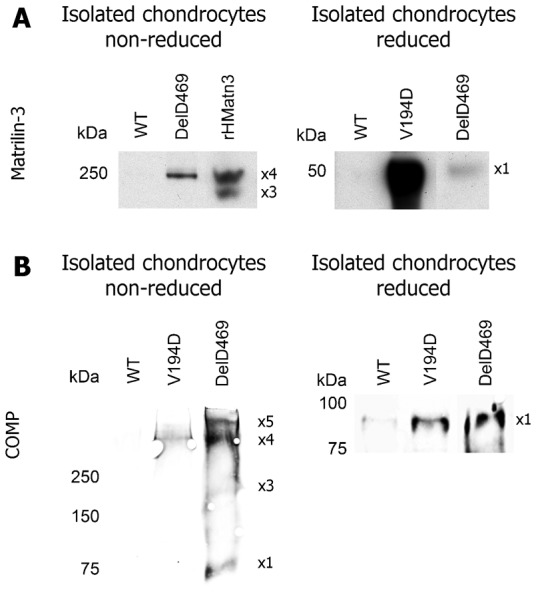
Wild type matrilin-3 is co-retained with mutant COMP in *Comp* DelD469 chondrocytes; wild type COMP is co-retained in the chondrocytes of *Matn3* V194D mice. Total protein lysate samples from isolated chondrocytes of wild type (WT) and mutant (*Matn3* V194D, *Comp* DelD469) cartilage were separated by SDS-PAGE (reduced or non-reduced conditions) and analyzed by Western blotting using antibodies specific to (**A**) matrilin-3 or (**B**) COMP. Key: ×1 = putative monomers; putative oligomeric forms of proteins (×2 = dimers, ×3 = trimers, ×4 = tetramers, ×5 = pentamers); rHMatn3 = recombinant full-length human matrilin-3 protein; kDa = mass in kilo Daltons.

We hypothesized that the wild type matrilin-3 extracted in buffer 1 from *Comp* DelD469 and *Comp* T585M samples might represent intracellular retained protein (Fig. 2B, top panel). We tested this hypothesis by performing SDS-PAGE and Western blotting of proteins isolated from the chondrocytes of 5-day-old mutant *Comp* mice. This analysis confirmed that wild type matrilin-3 was co-retained with mutant COMP in *Comp* DelD469 chondrocytes as a tetramer (Fig. 3A, DelD469 in left panel). However, the level of co-retention of wild type matrilin-3 in *Comp* DelD469 chondrocytes was low in comparison to the retention of mutant matrilin-3 in *Matn3* V194D chondrocytes (Fig. 3A, compare V194D with DelD469 in right panel). Furthermore, the retained wild type matrilin-3 was not present as high molecular weight aggregates in the *Comp* DelD469 chondrocytes (not shown). In contrast, we were not able to demonstrate the significant intracellular retention of matrilin-3 in isolated *Comp* T585M chondrocytes (not shown), which is consistent with the T585M form of mutant COMP being efficiently secreted (Piróg-Garcia et al., 2007). This latter finding therefore suggests that increased matrilin-3 extraction with buffer 1 from *Comp* T585M is most likely due to disrupted anchoring/interactions directly due to the T585M mutation in COMP.

### Wild type COMP is co-retained in the chondrocytes of *Matn3* V194D mice

Sequential protein extractions using buffers 1 and 2 indicated that COMP was more readily extracted from *Matn3* V194D cartilage than wild type cartilage (Fig. 2B, bottom panel) and we hypothesized that at least some of this COMP could represent intracellular co-retained protein. We therefore analyzed proteins extracted from isolated chondrocytes of *Matn3* V194D cartilage and free from any contaminating ECM. Wild type COMP was detected within the chondrocytes of *Matn3* V194D mice but at a lower concentration than the mutant COMP detected in *Comp* DelD469 chondrocytes (Fig. 3B). Under non-reducing conditions the retained wild type COMP within *Matn3* V194D chondrocytes appeared as a single high molecular weight oligomeric form (>250 kDa), which was consistent with a tetrameric form of COMP (Fig. 3B, V194D in left panel). In contrast, several oligomeric forms of mutant COMP were detected in *Comp* DelD469 chondrocytes (Fig. 3B, DelD469 in left panel), which could be resolved to monomers under reducing conditions (Fig. 3B, right panel).

### The adoption of a non-biased proteomic method to compare the mouse cartilage protein extractions confirms genotype specific differences in extractability

Given that it was not practical to analyze the extraction profiles for every cartilage ECM component using our candidate approach, we used a semi-quantitative proteomic method to compare cartilage protein extractions in a global and non-biased manner. For this investigation we used a method of in-gel trypsin digestion of total proteins sequentially extracted from mouse cartilage followed by LC-MS/MS analysis. In order to quantify the relative levels of each protein identified we used spectral counting, which has been established as a valid method for this approach (Liu et al., 2004; Lundgren et al., 2010).

We compared the relative quantities of peptide spectra that were detected by LC-MS/MS in wild type, *Matn3* V194D, *Comp* DelD469 and *Comp* T585M cartilage extracted by buffers 1, 2 and 3. The number of spectra identified for each protein were compared between wild type and mutant genotypes using the beta binomial test (Pham et al., 2010). Table 1 summarizes the total number of proteins identified and the number of proteins that were significantly changed in quantity between mutant and wild type cartilage samples. The individual genes that encode the relevant proteins were also subjected to DAVID functional analysis in order to group proteins sharing common molecular functions, cellular compartment or biological processes into annotation clusters (see supplementary material Tables S1–S3 for a list of all proteins significantly changed between mutant and wild type cartilage). In the first instance we chose to focus on changes in the extraction profiles of cartilage ECM proteins.

**Table 1.**
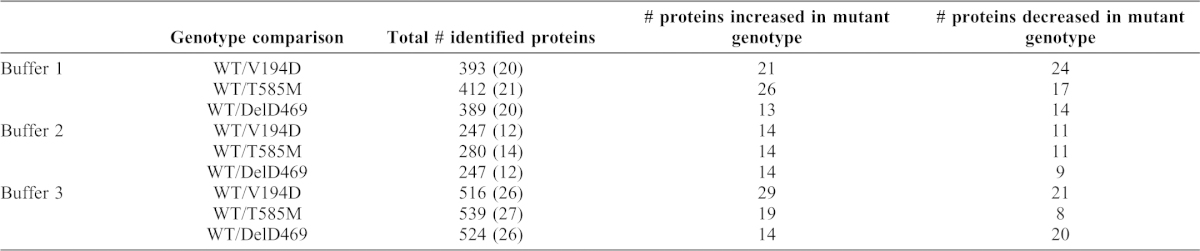
Summary of the results from LC-MS/MS analysis of cartilage sequential extractions. The number of proteins identified and the numbers of proteins significantly (*P*<0.05) increased/decreased in quantity in mutant samples compared with wild type controls are indicated. The predicted number of results falsely identified as being significant by the beta-binomial test is indicated in brackets for each comparison.

### The identification of common and discrete ECM disease signatures in PSACH-MED cartilage

**Fig. 4.**
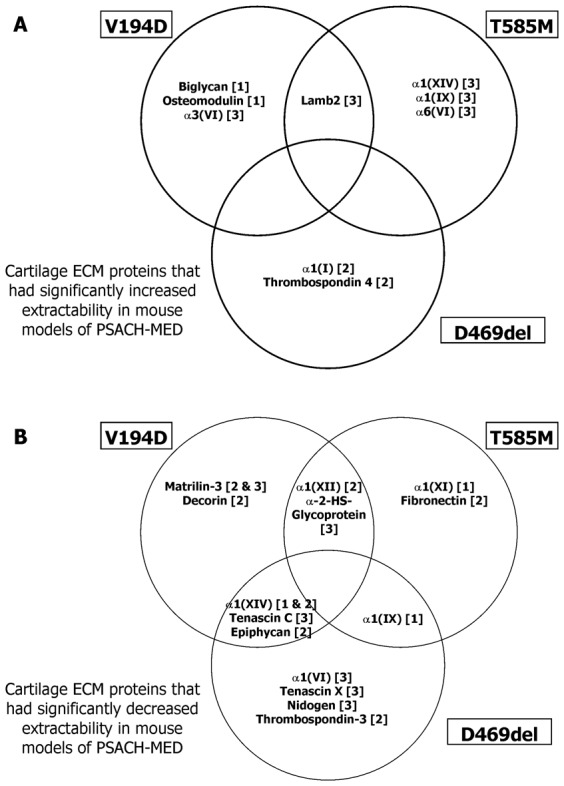
Genotype specific proteomic differences in mouse models of PSACH-MED. Schematic showing cartilage ECM proteins that had either significantly increased (**A**) or decreased (**B**) extractability in mouse models of *Matn3* V194D (V194D), *Comp* T585M (T585M) and *Comp* DelD469 (DelD469). Proteins with comparable extractability are shown in the overlapping areas and the numbers in square brackets indicate which specific buffers the proteins were extracted with.

In order to identify both commonalities and differences in the extraction profiles of structural ECM components from the three different mouse models of PSACH-MED, we compared the different MS-derived peptide profiles (unweighted spectra number) (supplementary material Tables S1–S3) from mice of all three genotypes (Fig. 4).

We noted common extraction profiles for two FACIT (fibril associated collagens with interrupted triple helices) collagen polypeptide chains; α1(XII) had decreased extractability in *Matn3* V194D and *Comp* T585M cartilage with buffer 2, whilst α1(XIV) had decreased extractability in *Matn3* V194D and *Comp* DelD469 cartilage with buffers 1 and 2 (Fig. 4B). In contrast α1(XIV) had increased extractability with buffer 3 from *Comp* T585M cartilage (Fig. 4A).

Interestingly, in *Comp* T585M cartilage α1(IX) had marginally decreased extractability in buffer 1, but considerably increased extractability in buffer 3, which partially validated the observations from the SDS-PAGE analysis (Fig. 1). Surprisingly, the only significant change in type IX collagen extractability in cartilage from *Comp* DelD469 mice was a slight decrease in buffer 1; whilst a trend towards increased extractability from buffer 3 samples was observed (*P* = 0.072), even though there had been clear differences in SDS-PAGE analysis of buffer 3 samples. Overall these observations suggest that important interactions between COMP/matrilin-3 and various FACIT collagens (types IX, XII and XIV) are disrupted by the PSACH-MED mutations.

Tenascin X was noticeably less extractable from *Comp* DelD469 cartilage (buffer 3), whilst Tenascin C was less extractable from both *Comp* DelD469 and *Matn3* V194D cartilage (buffer 3) (Fig. 4B), suggesting an important role for tenascin proteins in disrupted cell–matrix interactions within these mutant cartilages. Finally, alpha-2-HS-glycoprotein (fetuin-A), which has anti-calcification properties was less extractable from both *Matn3* V194D and *Comp* T585M cartilage (buffer 3) (Fig. 4B).

In addition to the common profiles, we also noted unique ECM extraction profiles for *Matn3* V194D [matrilin-3 and decorin], *Comp* T585M [α1(XI) and fibronectin] and *Comp* DelD469 [α1(VI), nidogen, thrombospondin-3 and tenascin X] mice, suggesting that genotype specific differences in cartilage ECM organization may also contribute to disease pathogenesis (Fig. 4B).

In contrast to ECM proteins with decreased extractability, the only common profile for increased extractability was for laminin β2 in *Matn3* V194D and *Comp* T585M cartilage (buffer 3), whilst unique extraction profiles were noted for *Matn3* V194D [the SLRPs biglycan and osteomodulin and also α3(VI)], *Comp* T585M [α6(VI), α1(IX) and α1(XIV)] and *Comp* DelD469 [α1(I) and thrombospondin-4] cartilage samples (Fig. 4A).

### Validation of ECM disease signatures by targeted Western blot analysis

**Fig. 5.**
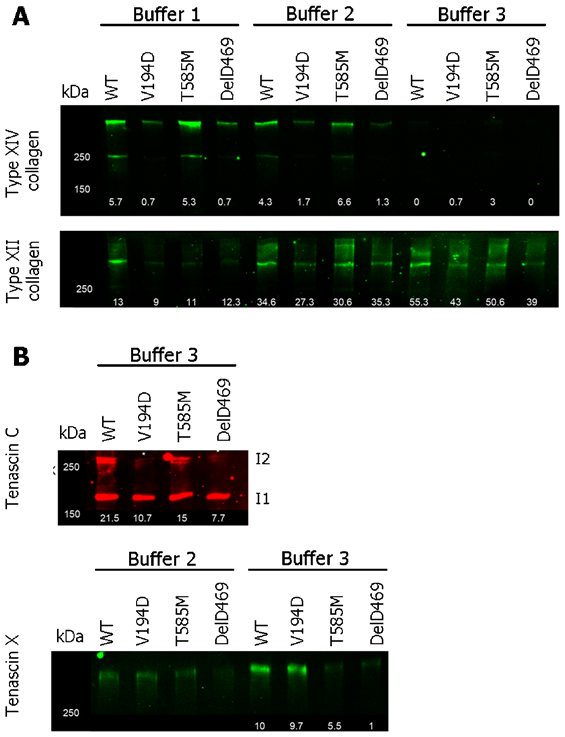
Validation of proteomic analysis by SDS-PAGE and Western blotting. SDS-PAGE and Western blot analysis of sequential cartilage extractions for validation of the proteomic approach. Cartilage from wild type (WT) and mutant (*Matn3* V194D, *Comp* T585M and *Comp* DelD469) mice was extracted in a series of three buffers (Buffer 1–3) and separated under reducing conditions. (**A**) Analysis of types XII and XIV FACIT collagens, (**B**) analysis of tenascin C and tenascin X. Results are shown for every buffer where protein was detected by Western blot analysis. Mouse genotypes are indicated at the top of each lane and the average numbers of matched peptide spectra detected by LC-MS/MS analysis of the same cartilage extractions are shown at the bottom of each lane. Key: I1 and I2 indicate the sizes of the different isoforms of tenascin C; kDa = mass in kilo Daltons.

The spectral count data were verified using SDS-PAGE and Western blot analyses of the cartilage protein extractions (Fig. 5). For this validation of the LC-MS/MS approach we chose to focus only on those ECM proteins that were commonly decreased in extractability in more than one mouse model (e.g. types XIV and XII collagen and tenascin C), or highly decreased in extractability in a single mouse model (tenascin X in DelD469 COMP) (Fig. 2; supplementary material Tables S1–S3).

Following SDS-PAGE and Western blotting the band intensities of proteins largely mirrored the spectral count data derived from mass spectrometry analyses. For example, there was a clear reduction in type XIV in protein samples from *Matn3* V194D and *Comp* DelD469 cartilage (buffers 1 and 2) (Fig. 5A, top panel) that closely correlated with the LC-MS/MS data (supplementary material Tables S1–S3). Type XII collagen reactivity was also correspondingly reduced in *Matn3* V194D samples (buffer 2), but it was also clear that there were other changes in extractability using buffers 1 and 3 that had not been detected using LC-MS/MS (Fig. 5A, bottom panel). In particular, type XII collagen was less extractable in buffer 1 from the cartilage of all mutant mice.

Analysis of tenascin C indicated that it was specifically the largest protein isoform (I2: ∼250 kDa) that was reduced in *Matn3* V194D and *Comp* DelD469 buffer 3 extractions when compared with wild type and *Comp* T585M cartilages (Fig. 5B, top panel). In addition, reduced extractability of tenascin X was detected in cartilage from both the *Comp* T585M (buffer 3 only) and *Comp* DelD469 (buffers 2 and 3) mice (Fig. 5B, bottom panel) that was slightly different to spectral count data, which had only revealed a significant decrease in extractability of tenascin X in *Comp* DelD469 buffers 3 samples. There was no difference in the extraction of tenascin X from wild type and *Matn3* V194D cartilages, which was consistent with LC-MS/MS data.

### Proteomic validation of the intracellular ‘stress proteome’ disease signature in matrilin-3 V194D chondrocytes

In addition to identifying changes in the extractability of cartilage ECM proteins we were also interested to know if the proteomic-derived data correlated with the disease specific signatures previously defined using gene expression studies. For this comparison we compared the proteome from *Matn3* V194D cartilage (supplementary material Table S1) with previously published gene array data for this mouse model (Nundlall et al., 2010). The LC-MS/MS approach identified significantly increased protein levels of several chaperones and foldases including Grp94, Grp78 (BiP) and calreticulin (Calr), members of the protein disulphide isomerase family (PDIA 3, 4 and 6) and two novel ER-stress induced proteins, CRELD2 and Armet/Manf. Significantly, all these proteins were previously shown to be transcriptionally upregulated by microarray analysis (Nundlall et al., 2010). Conversely, the extraction of proteins associated with intracellular protein trafficking were significantly reduced in *Matn3* V194D samples (e.g. buffer 1: coatomer subunit beta (COPB2), coatomer subunit delta (COPD) and GTPase Ran) (supplementary material Table S1), which is consistent with the intracellular retention of mutant matrilin-3 (Fig. 3). Furthermore, the amount of proliferating nuclear cell antigen (PNCA) was decreased in *Matn3* V194D samples, concomitant with the decreased rate of chondrocyte proliferation previously observed in these mutant mice (Leighton et al., 2007).

## Discussion

In this study we investigated the cartilage protein extraction profile of three gene targeted mouse models of phenotypically-related chondrodysplasias using both candidate and global approaches in order to gain insight into ECM disorganization and derive new perspectives on disease mechanisms and cartilage degradation. This is the first time that such a dual approach has been used to analyse cartilage from genetic models of human ECM diseases.

SDS-PAGE and Western blot analysis of sequentially extracted knee cartilage revealed genotype-specific differences in the extraction of a number of proteins that included matrilin-1 to -4, COMP and type IX collagen, which are all known to interact with each other (Budde et al., 2005; Fresquet et al., 2010; Fresquet et al., 2007; Holden et al., 2001; Mann et al., 2004; Wiberg et al., 2003). While we were unable to analyse the insoluble material that remained following extraction of cartilage tissue (and therefore could not quantify total differences in protein abundance), our method was especially useful in allowing us to identify subtle differences in the extraction of individual protein oligomers that may not have been detected using other methods. For example, two specific (hetero)oligomeric forms of matrilin-1 were clearly absent from *Matn3* V194D cartilage extractions and given our previous data confirming the intracellular co-retention of a proportion of wild type matrilin-1 with mutant matrilin-3 in the *Matn3* V194D model (Bell et al., 2012), we hypothesize that these matrilin-1 oligomers missing from *Matn3* V194D cartilage extractions represent matrilin-1/-3 hetero-oligomers, which we propose are present within the heterogeneous disulphide bonded aggregates retained within the ER of *Matn3* V194D chondrocytes.

We detected a form of type IX collagen in sequential extractions from *Comp* DelD469 and *Comp* T585M cartilage that contained the α1(IX) NC4 domain and which corresponded in size to the predicted molecular weight of the α1(IX) chain. This form of type IX collagen was not extractable using the same conditions from wild type or *Matn3* V194D cartilage. Robust microarray studies and immunohistochemical analyses have previously demonstrated that there are comparable levels of type IX collagen gene expression (Nundlall et al., 2010; Suleman et al., 2012) and protein between the cartilage of wild type and all three mutant mice (Leighton et al., 2007; Piróg-Garcia et al., 2007; Suleman et al., 2012) confirming that we had indeed detected differences in extractability, rather than genotype specific differences in the relative amounts of type IX collagen. The results from LC-MS/MS analysis partially validated our finding of increased α1(IX) collagen extraction from *Comp* T585M and *Comp* DelD469 cartilage. For example, a significant increase in the quantity of α1(IX) collagen was detected in *Comp* T585M buffer 3 samples relative to wild type controls and a trend towards an increase in α1(IX) collagen extraction was observed in *Comp* DelD469 buffer 3 samples, but this did not reach statistical significance (WT spectra = 2 vs DelD469 spectra = 5; *P* = 0.072; data not shown). However, in contrast to Western blot analysis which detected no differences in the extraction of type IX collagen from *Comp* DelD469 buffer 1 samples, LC-MS/MS revealed a small decrease from similar extractions (WT spectra = 2 vs DelD469 spectra = 0; *P* = 0.019). The small number of matched spectra implies that this could potentially represent a false positive, and therefore demonstrates the importance of using complementary methods to gain an accurate understanding of the cartilage proteome.

Types II, IX and XI collagen are highly cross-linked in mouse cartilage by 3 weeks of age (Mendler et al., 1989); therefore, the identification of extractable type IX collagen from *Comp* DelD469 and *Comp* T585M cartilage suggests that it may be less tightly integrated into the ECM of this abnormal cartilage. This would have an impact upon the integrity of the cartilage ECM and given the known function of type IX collagen in cartilage and its influence on cartilage degradation when knocked out (Fässler et al., 1994), may ultimately contribute to the early onset OA seen in PSACH and MED patients.

We also identified differences in the molecular forms of matrilin-3 that were extracted from *Comp* T585M and *Comp* DelD469 cartilage by buffer 1. The matrilin-3 form that was absent from *Comp* DelD469 samples was identified in wild type (following longer exposure, data not shown) and *Comp* T585M buffer 1 extractions and we hypothesize that this oligomer may correspond to an N-terminally-cleaved form of tetrameric matrilin-3 (Hills et al., 2007). This observation suggests a possible difference in matrilin-3 processing in *Comp* DelD469 mice, the pathological consequence of which is not known. Interestingly, matrilins are cleaved by ADAMT4 and -5 (Ehlen et al., 2009) and it was postulated that the release of matrilin-3 from the matrix could in turn lead to further induction of pro-inflammatory cytokines and proteases in chondrocytes (Klatt et al., 2009) which could influence the occurrence of OA at later stages of PSACH-MED.

The finding of an overall increase in the extractability of matrilin-3 and COMP by buffer 1 in all three PSACH-MED mouse models led us to consider that some of this protein could represent intracellular retained material. Analysis of protein lysates from isolated chondrocytes confirmed this hypothesis for the *Matn3* V194D and *Comp* DelD469 models, but more importantly, it also confirmed that whilst both mutant proteins are retained, only mutant V194D matrilin-3 is retained as a high molecular weight covalently-linked aggregate (Bell et al., 2012). This important observation demonstrates that the consequences of the individual mutations on protein misfolding and retention/accumulation differs between *Matn3* V194D and *Comp* DelD469 and perhaps such differences begin to provide a molecular explanation for why, in contrast to *Matn3* V194D, *Comp* DelD469 does not elicit a classical unfolded protein response (Suleman et al., 2012).

We also used a proteomic method (LC-MS/MS spectral counting) to compare cartilage sequential protein extractions in a global non-biased manner. Our investigation made use of the same cartilage extraction protocol used for Western blot analysis and we detected a total of 957 unique proteins, which was comparable with previous murine cartilage proteomic investigations (Belluoccio et al., 2006; Pecora et al., 2007). LC-MS/MS analysis of femoral cartilage extractions identified significant changes in the extraction profiles of many proteins including structural and non-structural ECM proteins, in addition to proteins with known roles in protein folding and trafficking.

Among the most consistent findings was a relative decrease in the amount of type XIV collagen extracted from *Matn3* V194D and *Comp* DelD469 cartilage compared with control and *Comp* T585M samples. This decrease was evident through both spectral counting and Western blotting approaches. Type XIV collagen has recently been identified as a binding partner of COMP *in vitro* (Agarwal et al., 2012), which suggests the possibility that a disruption to the ECM by *Matn3* V194D and *Comp* DelD469 mutations could affect type XIV collagen stability in the ECM by altering direct binding between these proteins. This finding is especially important given the role of type XIV in collagen fibrillogenesis (Ansorge et al., 2009), the known disruption to collagen fibrillogenesis caused by an MED-causing COMP mutation (Hansen et al., 2011) and the previous finding of altered collagen fibril morphology in *Matn3* V194D, *Comp* DelD469 and *Comp* T585M cartilages (Leighton et al., 2007; Piróg-Garcia et al., 2007; Suleman et al., 2012) (supplementary material Fig. S3).

Interestingly, and somewhat in contrast to type XIV collagen, there was decreased extraction of type XII collagen from *Matn3* V194D and *Comp* T585M cartilages based on LC-MS/MS analysis of buffer 2 samples. This observation was partially validated by Western blotting, although the latter technique identified additional differences that had not been detected by LC-MS/MS. Recently, type XII collagen has been shown *in vitro* to bind COMP via its collagenous domains (Agarwal et al., 2012). Taken together these findings suggest a close functional relationship between glycoproteins (matrilin-3 and COMP) and FACIT collagens (Types IX, XII and XIV) in the chondrocyte pericellular matrix and that disruptions to this network might be a key disease trigger in cartilage degradation. Indeed, electron microscopy of cartilage from the three mouse models has previously demonstrated changes in the morphology of the ECM (Leighton et al., 2007; Piróg-Garcia et al., 2007; Suleman et al., 2012). In particular, the collagen fibrils were more clearly visible, suggesting that lower levels of fibril surface-associated proteins were decorating individual collagen fibrils (supplementary material Fig. S3). The common and discrete disease signatures that we have identified in this study might therefore explain these differences in the ‘cartilage phenotype’ of these genetic mouse models of PSACH-MED.

A further difference in the extraction of a collagen-interacting protein was observed in *Comp* DelD469 cartilage extractions, where there was a significant decrease in the detection of tenascin X compared with other genotypes. Tenascin X has only recently been detected in cartilage (Wilson et al., 2012) and its knockout in mice has been shown to cause a reduction in the collagen content of skin (Mao et al., 2002). Furthermore, a wide variety of gene deletion and truncating point mutations lead to tenascin X deficiency in skin and recessive Ehlers–Danlos syndrome (Schalkwijk et al., 2001); a disease characterized by hyperextensible skin, hypermobile joints and tissue fragility. The knockout of tenascin X in mice, and its deletion in humans, also causes alterations in muscle function (Huijing et al., 2010; Voermans et al., 2011) that induces histological features of a mild myopathy, which is also observed in *Comp* mutant mice (Piróg et al., 2010). Our finding provides the first evidence of changes in the extractability of tenascin X from the cartilage of a chondrodysplasia disease model. If the reduced detection of tenascin X in cartilage were to extrapolate to other tissues where mutant COMP is expressed this could potentially have pathological consequences and contribute to the myotendonopathy associated with PSACH and MED (Piróg and Briggs, 2010).

A notable and potentially related observation was the decreased detection of tenascin C in *Matn*3 V194D and *Comp* DelD469 cartilage extractions. Tenascin C is an extracellular glycoprotein involved in tissue injury/repair and is upregulated in osteoarthritic cartilage, where tenascin C protein fragments are believed to induce inflammatory mediators and ECM degradation (Patel et al., 2011; Sofat et al., 2012). We found no evidence of tenascin C fragmentation in *Matn*3 V194D and *Comp* DelD469 samples since the examination of matched peptides did not reveal any genotype specific differences in sequence coverage (data not shown). Interestingly, the decrease in tenascin C in *Comp* DelD469 samples was accompanied by a corresponding reduction in transforming growth factor beta induced protein ig-h3 (Tgfbi) (supplementary material Table S3). Both of these proteins have recently been proposed to contribute to TGFβ1 signaling in early cartilage development (Wilson et al., 2012) and alterations within TGFβ1 signaling have been linked to osteoarthritis (van der Kraan et al., 2010), whilst *COMP* itself has recently been identified as a TGFβ-responsive gene (Li et al., 2011). The detrimental effects of chondrodysplasia-causing mutations on the TGFβ1 signaling pathway could therefore yield pathological consequences for cartilage development and degeneration and potentially contribute to PSACH and MED disease mechanisms. Alternatively, a decrease in TGFβ1 signaling in *Comp* DelD469 cartilage might form part of a feedback mechanism to reduce the expression of mutant COMP.

The LC-MS/MS approach also validated the intracellular disease signature of *Matn3* V194D chondrocytes by confirming increased protein levels of specific chaperones (Armet, Creld2, Grp78, Grp94 and Calr) and foldases (PDIA 3, 4 and 6), that have been previously shown to be highly upregulated at the transcriptional level (Nundlall et al., 2010). These transcriptional changes therefore support the proteomic analyses described in this investigation.

Finally, recent studies have shown that numerous myogenic genes (including *MYH4*) are expressed in articular cartilage, and that changes in their expression constitutes a novel disease signature in the STR/Ort murine model of OA (Poulet et al., 2012). Interestingly, increased levels of myosin heavy polypeptide 4 (MYH4) were detected in *Matn3* V194D, *Comp* T585M and *Comp* DelD469 cartilage (buffer 2) compared with controls (supplementary material Tables S1–S3). These data therefore provide new insight into the cartilage degeneration that is associated with PSACH and MED and identifies areas for further investigation.

To conclude, we have completed a proteomic analysis of *Matn3* V194D, *Comp* T585M and *Comp* DelD469 models of chondrodysplasia and demonstrated that the mutation of matrilin-3 or COMP can induce changes to the extractability of other cartilage proteins confirming that there are disruptions to the organization of the cartilage ECM. Whilst many of these changes in ECM organization are genotype specific, there is also likely to be a generalized disruption to cartilage integrity that ultimately increases susceptibility to osteoarthritis, which is common to both PSACH and MED. This semi-quantitative proteomic approach, in combination with gene expression studies, also provides a powerful tool to generate reference ‘-omics profiles’ that will help establish disease signatures in chondrodysplasias that are also relevant to more common forms of joint degeneration such as osteoarthritis. Defining these diseases signatures is a prerequisite for the identification and validation of potential biomarkers and future therapeutic applications.

## Materials and Methods

### Breeding of mice

*Matn3* V194D, *Comp* DelD469 and *Comp* T585M mice were generated as previously described (Leighton et al., 2007; Piróg-Garcia et al., 2007; Suleman et al., 2012) and control mice were of an equivalent mixed genetic background (50% C57BL/6 and 50% 129Sv) to all mutant lines. Wild type and mutant mice were used to provide cartilage for sequential protein extractions and chondrocytes for Western blot analysis of intracellular proteins.

### Cartilage protein extraction and chondrocyte isolation

Cartilage from knee joints and femoral heads were dissected from the hind limbs of 3-week-old mice and frozen at −80°C. Knee joints were dissected by removal of the patella tendon and other connective tissue surrounding the joint using a scalpel, followed by excision of all tissue proximal/distal to tibial/femoral epiphyseal growth plates, respectively. Femoral heads were dissected by careful removal from the acetabulum using a scalpel, followed by removal of all tissue distal to the femoral head. Cartilage samples were thawed and sequentially extracted in buffer 1 (0.15 M NaCl, 50 mM Tris [pH 7.4]), buffer 2 (1 M NaCl 10 mM EDTA, 50 mM Tris [pH 7.4]) and buffer 3 (4 M GuHCl, 10 mM EDTA, 50 mM Tris [pH 7.4]) as previously described (Nicolae et al., 2007). All buffers contained 2 mM phenylmethylsulfonyl fluoride and 2 mM *N*-ethylmaleimide protease inhibitors. The supernatants from the extractions were ethanol precipitated, air dried and resuspended as previously described (Nicolae et al., 2007). 20 µl aliquots of each extraction were prepared and frozen at −80°C prior to analysis by SDS-PAGE. Costal chondrocytes were isolated as previously described (Nundlall et al., 2010).

### SDS-polyacrylamide gel electrophoresis and immunoblotting

Aliquots from three biological replicates per genotype (i.e. tissue extracts from 3 different mice) of knee or femoral head cartilage extractions and rib chondrocyte preparations were thawed and separated on 4–13.5% and 4–12% SDS-polyacrylamide gels respectively. When protein samples were reduced, dithiothreitol (DTT) was added to a final concentration of 0.1 M and samples were boiled at 90°C for 5 min prior to loading. Proteins were transferred to nitrocellulose membranes and uniform transfer was evaluated by ponceau staining (supplementary material Fig. S4). The primary antibodies used were Matrilin-1 (Hauser and Paulsson, 1994), -2 (Piecha et al., 1999), -3 (reduced samples, R&D; non-reduced samples (Klatt et al., 2000)), -4 (Klatt et al., 2001), COMP (reduced samples, Genetex; non-reduced samples (DiCesare et al., 1994)), type IX collagen NC4 domain (Budde et al., 2005), type XII collagen (Veit et al., 2006), type XIV collagen (Ansorge et al., 2009), tenascin C (Sigma), tenascin X and decorin (Wiberg et al., 2003). Membranes were incubated with either peroxidase-conjugated or near-infrared fluorescently labeled secondary antibodies for 1 h. Secondary antibodies used were peroxidase-conjugated swine anti-rabbit IgG (Dako), donkey anti-mouse, anti-rabbit, anti-guinea pig and anti-goat IgGs (Licor).

Those blots incubated with fluorescently labeled antibodies were imaged using the Odyssey system (Licor). Peroxidase-conjugated antibodies were detected using 3-amin-opthalhydrazide (1.25 mM), *p*-coumaric acid (225 µM) and 0.01% H_2_O_2_ then exposed to photographic film.

### Mass spectrometry (MS) analysis of cartilage

20 µl aliquots of femoral head cartilage extracts were run on 4–12% SDS-polyacrylamide gels for 4 mins (at 200 V). Total protein pools were excised from the gel before being dehydrated, reduced, alkylated and washed. Samples were then digested with trypsin overnight at 37°C and analysed by LC-MS/MS using a NanoAcquity LC (Waters, Manchester, UK) coupled to a LTQ Velos (Thermo Fisher Scientific, Waltham, MA) mass spectrometer. Peptides were concentrated on a pre-column (20 mm×180 µm i.d, Waters). The peptides were then separated using a gradient from 99% A (0.1% formic acid in water) and 1% B (0.1% formic acid in acetonitrile) to 25% B, in 45 min at 200 nL min^−1^, using a 75 mm×250 µm i.d. 1.7 µM BEH C18, analytical column (Waters). Peptides were selected for fragmentation automatically by data dependent analysis.

### Bioinformatic processing of proteomics data

Data were interrogated using Mascot version 2.2 (Matrix Science, UK) against the UniProt database (version 2011-05) with taxonomy of *Mus musculus* and the following search parameters selected: fragment tolerance: 0.6 Da; parent tolerance: 0.5 Da; fixed modifications allowed: +57 on C (carbamidomethyl), +16 on M (oxidation); max missed cleavages: 1. Mascot search results were validated using Scaffold version 3.3.1 (Proteome Software, Portland, USA) to assign confidence values to peptide/protein matches, where Peptide/Protein Prophet algorithm confidence values of 0.7 and 0.99 were used respectively. Identified proteins were defined as having a number of matched peptide spectra ≥2, and the unweighted spectral count was used as a measure of quantification. These parameters constrained the protein false discovery rate (FDR) to ≤0.2% in all analyses. Three biological replicates were used in all experiments except for the analysis of buffer 3 wild type samples, where two biological replicates were used (as a result of sample failure in one replicate). The number of spectra identified for each protein were compared between wild type and mutant genotypes using the beta-binomial test in R (version 2.14.2, BetaBinomial package) (Pham et al., 2010). A *P*-value<0.05 was considered significant. Lists containing the Uniprot IDs of proteins that were significantly different in quantity between wild type/mutant samples were analysed using The Database for Annotation, Visualization and Integrated Discovery (DAVID) bioinformatics resource 6.7 (http://david.abcc.ncifcrf.gov) (Dennis et al., 2003).

## Supplementary Material

Supplementary Material
