# Supplementary material for: Analysis of the cartilage proteome from three different mouse models of genetic skeletal diseases reveals common and discrete disease signatures
Source: Biol Open. 2013 Jun 18;2(8):802–11. doi: 10.1242/bio.20135280 (PMC3744072; doi:10.1242/bio.20135280)
Supplement: Supplementary Material [file supp_2_8_802_v2_index.html]

Analysis of the cartilage proteome from three different mouse models of genetic skeletal diseases reveals common and discrete disease signatures — Analysis of the cartilage proteome from three different mouse models of genetic skeletal diseases reveals common and discrete disease signatures — Supplementary Material 

# Analysis of the cartilage proteome from three different mouse models of genetic skeletal diseases reveals common and discrete disease signatures

## 

**Files in this Data Supplement:**

- Supplementary Material - Peter A. Bell et al. doi: 10.1242/bio.20135280
- Supplementary Tables
