# Supplementary material for: Analysis of the cartilage proteome from three different mouse models of genetic skeletal diseases reveals common and discrete disease signatures
Source: Biol Open. 2013 Jun 18;2(8):802–11. doi: 10.1242/bio.20135280 (PMC3744072; doi:10.1242/bio.20135280)
Supplement: Supplementary Material [file supp_bio.20135280_bio.20135280-s1.pdf]

Biology Open

Supplementary Material

Peter A. Bell et al. doi: 10.1242/bio.20135280

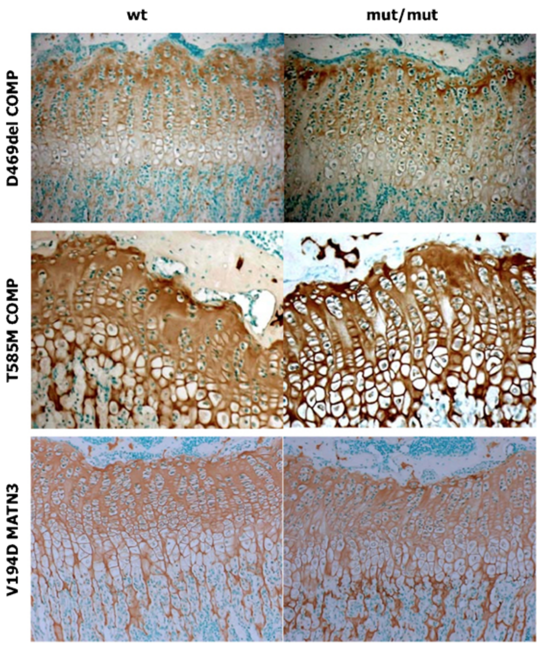

Fig. S1. Immunohistochemistry confirms comparable levels of type IX collagen in the cartilage growth plates of mouse models of PSACH-MED. Representative images of the growth plates of 3-week-old wild-type and mutant mice (*Matn*3 V194D, *Comp* D469 del and *Comp* T585M) stained with an antibody raised against type IX collagen.

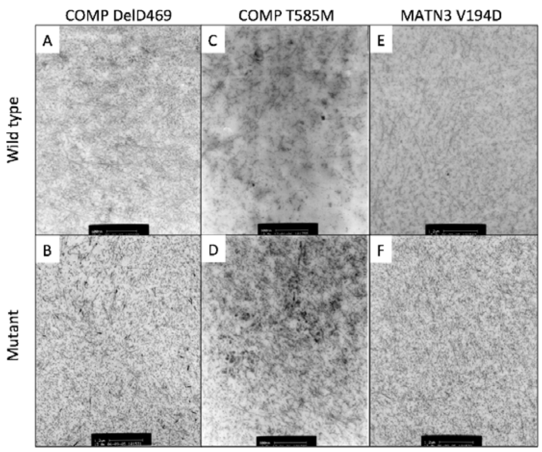

Fig. S3. Transmission electron microscopy of cartilage from 1-week-old mice models of PSACH and MED. Representative TEM images of the knee cartilage of 1-week-old wild-type and mutant mice; (**A**,**B**) COMP DelD469, (**C**,**D**) COMP T585M and (**E**,**F**) MATN3 V194D. The collagen fibrils were more clearly visible in mutant cartilage suggesting that lower levels of fibril surface-associated proteins were decorating individual collagen fibrils. Scale bars: 600nm (A), 1.2µm (B), 800nm (C), 800nm (D), 1.2µm (E), 1.2µm (F).

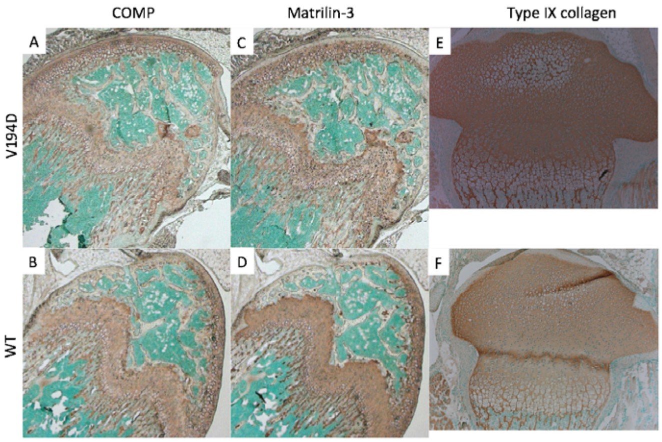

Fig. S2. Immunohistochemistry confirms comparable levels of type IX collagen in the knee cartilage of a mouse model of Matn3 MED. Representative images of the knee cartilage of 3-week-old wild-type and mutant mice (*Matn*3 V194D) stained with antibodies raised against COMP (**A**,**B**), matrilin-3 (**C**,**D**) and 1-week-old mouse stained with type IX collagen (**E**,**F**).

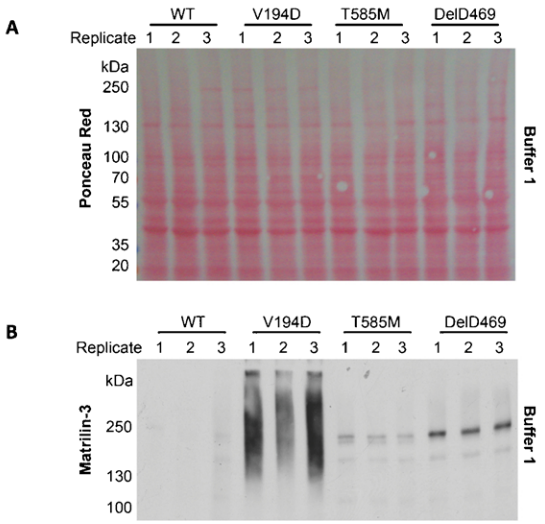

Fig. S4. Loading controls and reproducibility of Western blot analyses of sequential protein extraction of knee joint cartilage. Representative images of cartilage extracted with buffer 1 (3 biological replicate samples per genotype: WT, *Matn*3 V194D, *Comp* D469del and *Comp*T585M) separated by SDS-PAGE, transferred to nitrocellulose membrane and (**A**) stained with ponceau red as a loading control and (**B**) probed with an antibody raised against matrilin-3.
