# Supplementary material for: Analysis of the cartilage proteome from three different mouse models of genetic skeletal diseases reveals common and discrete disease signatures
Source: Biol Open. 2013 Jun 18;2(8):802–11. doi: 10.1242/bio.20135280 (PMC3744072; doi:10.1242/bio.20135280)
Supplement: Supplementary Material [file supp_bio.20135280_Tables_S1-S3.docx]

| **Protein** | **Uniprot ID** | **WT spectra number** | **V194D spectra number** | **p value** | **ER lumen (CC)** | **GAG binding (MF)** | **PDI activity (MF)** | **Ribonucleotide binding**  **(MF)** |
| --- | --- | --- | --- | --- | --- | --- | --- | --- |
| heat shock protein 90, beta (Grp94), member 1 | ENPL_MOUSE | 9 | 25.67 | 0.0002 |  |  |  |  |
| cysteine-rich with EGF-like domains 2 | CREL2_MOUSE | 0 | 4 | 0.0005 |  |  |  |  |
| protein disulfide isomerase associated 4 | PDIA4_MOUSE | 4.67 | 13.33 | 0.0010 |  |  |  |  |
| heat shock protein 5 | GRP78_MOUSE | 9.67 | 22.67 | 0.0041 |  |  |  |  |
| protein disulfide isomerase | PDIA1_MOUSE | 5.33 | 12 | 0.0056 |  |  |  |  |
| protein disulfide isomerase associated 3 | PDIA3_MOUSE | 9.33 | 17.67 | 0.0060 |  |  |  |  |
| phosphoglucomutase 1 | PGM1_MOUSE | 0.67 | 4 | 0.0093 |  |  |  |  |
| aldo-keto reductase family 1, member A4 (aldehyde reductase) | AK1A1_MOUSE | 0.67 | 4 | 0.0093 |  |  |  |  |
| Nucleoside diphosphate kinase B (NDK B) | NDKB_MOUSE | 0.67 | 3.33 | 0.0210 |  |  |  |  |
| phosphoenolpyruvate carboxykinase 2 (mitochondrial) | PCKGM_MOUSE | 0 | 1.67 | 0.0218 |  |  |  |  |
| D-3-phosphoglycerate dehydrogenase | SERA_MOUSE | 0 | 2 | 0.0221 |  |  |  |  |
| mesencephalic astrocyte-derived neurotrophic factor | MANF_MOUSE | 0 | 1.67 | 0.0223 |  |  |  |  |
| glutamate dehydrogenase 1; predicted gene 5902 | DHE3_MOUSE | 0 | 1.33 | 0.0231 |  |  |  |  |
| pyrophosphatase (inorganic) 1 | IPYR_MOUSE | 0 | 2.67 | 0.0245 |  |  |  |  |
| complement factor B | CFAB_MOUSE | 0 | 1.33 | 0.0251 |  |  |  |  |
| ATP synthase, H+ transporting, mitochondrial F1 complex, alpha subunit, isoform 1 | ATPA_MOUSE | 0.67 | 3.33 | 0.0274 |  |  |  |  |
| serine (or cysteine) peptidase inhibitor, clade C (antithrombin), member 1 | ANT3_MOUSE | 0 | 1.33 | 0.0321 |  |  |  |  |
| serine (or cysteine) peptidase inhibitor, clade E, member 2 | GDN_MOUSE | 0 | 1.33 | 0.0321 |  |  |  |  |
| phosphoglycerate kinase 1 | PGK1_MOUSE | 5 | 9.67 | 0.0430 |  |  |  |  |
| Biglycan | PGS1_MOUSE | 0.67 | 2.67 | 0.0491 |  |  |  |  |
| Osteomodulin | OMD_MOUSE | 0.67 | 2.67 | 0.0499 |  |  |  |  |

**Table S1.**

Proteins significantly increased in quantity in *Matn3* V194D cartilage extractions (buffer 1) relative to the wild type (WT) control. Output from DAVID analysis is summarized by functionally related terms where (CC) denotes cellular compartment and (MF) denotes molecular function clusters; black boxes indicate membership of a grouping. Statistical significance was determined using the beta-binomial test and p<0.05 was considered significant.

| **Protein** | **Uniprot ID** | **WT spectra number** | **V194D spectra number** | **p value** | **ER lumen (CC)** | **Nucleotide binding (MF)** | **Carbohydrate binding (MF)** |
| --- | --- | --- | --- | --- | --- | --- | --- |
| protein disulfide isomerase associated 6 | PDIA6_MOUSE | 0 | 6.33 | 0.0002 |  |  |  |
| protein disulfide isomerase associated 4 | PDIA4_MOUSE | 0.67 | 8.67 | 0.0006 |  |  |  |
| serine (or cysteine) peptidase inhibitor, clade E, member 2 | GDN_MOUSE | 0 | 3 | 0.0012 |  |  |  |
| ribonuclease, RNase A family 4 | RNAS4_MOUSE | 0 | 2.67 | 0.0017 |  |  |  |
| heat shock protein 90, beta (Grp94), member 1 | ENPL_MOUSE | 6.33 | 18.67 | 0.0023 |  |  |  |
| heat shock protein 5 | GRP78_MOUSE | 6 | 15 | 0.0055 |  |  |  |
| high-mobility group (nonhistone chromosomal) protein 1-like 1 | HMGB1_MOUSE | 0 | 3 | 0.0228 |  |  |  |
| lysozyme 1 | LYZ1_MOUSE | 0 | 1.33 | 0.0262 |  |  |  |
| succinate dehydrogenase complex, subunit A, flavoprotein (Fp) | DHSA_MOUSE | 0 | 1.33 | 0.0297 |  |  |  |
| coagulation factor IX | FA9_MOUSE | 0 | 1.33 | 0.0297 |  |  |  |
| hypoxia up-regulated 1 | HYOU1_MOUSE | 0 | 1.33 | 0.0297 |  |  |  |
| poly(A) binding protein, cytoplasmic 1 | PABP1_MOUSE | 0 | 1.33 | 0.0297 |  |  |  |
| calreticulin | CALR_MOUSE | 1 | 4.33 | 0.0344 |  |  |  |
| myosin, heavy polypeptide 4, skeletal muscle | MYH4_MOUSE | 0 | 3 | 0.0468 |  |  |  |

Proteins significantly increased in quantity in *Matn3* V194D cartilage extractions (buffer 2) relative to wild type (WT) control. Output from DAVID analysis is summarized by functionally related terms where (CC) denotes cellular compartment and (MF) denotes molecular function clusters; black boxes indicate membership of a grouping. Statistical significance was determined using the beta-binomial test and p<0.05 was considered significant.

| **Protein** | **Uniprot ID** | **WT spectra number** | **V194D spectra number** | **p value** | **membrane bound vesicle (CC)** | **ER reticulum (CC)** | **organelle lumen (CC)** | **ribosome (CC)** | **nucleotide binding (MF)** |
| --- | --- | --- | --- | --- | --- | --- | --- | --- | --- |
| 78 kDa glucose-regulated protein | GRP78_MOUSE | 17.5 | 56.33 | 0.0003 |  |  |  |  |  |
| heat shock protein 90, beta (Grp94), | ENPL_MOUSE | 11 | 32.00 | 0.0003 |  |  |  |  |  |
| poly(rC) binding protein 1 | PCBP1_MOUSE | 0 | 3.00 | 0.0029 |  |  |  |  |  |
| calumenin | CALU_MOUSE | 0 | 3.67 | 0.0029 |  |  |  |  |  |
| hypoxia up-regulated 1 | HYOU1_MOUSE | 0 | 2.33 | 0.0065 |  |  |  |  |  |
| heat shock protein 4 | HSP74_MOUSE | 0 | 2.33 | 0.0066 |  |  |  |  |  |
| elastin microfibril interfacer 1 | EMIL1_MOUSE | 0 | 2.33 | 0.0070 |  |  |  |  |  |
| phosphoglycerate mutase 1 | PGAM1_MOUSE | 0 | 2.33 | 0.0069 |  |  |  |  |  |
| Bifunctional aminoacyl-tRNA synthetase | SYEP_MOUSE | 0 | 2.33 | 0.0069 |  |  |  |  |  |
| protein disulfide isomerase associated 4 | PDIA4_MOUSE | 5 | 12.67 | 0.0075 |  |  |  |  |  |
| serine (or cysteine) peptidase inhibitor, clade H, member 1 | SERPH_MOUSE | 21 | 38.00 | 0.0102 |  |  |  |  |  |
| protein disulfide isomerase associated 6 | PDIA6_MOUSE | 4.5 | 11.67 | 0.0104 |  |  |  |  |  |
| ribosomal protein S9 | RS9_MOUSE | 0 | 2.33 | 0.0317 |  |  |  |  |  |
| retinoblastoma binding protein 7 | RBBP7_MOUSE | 0 | 1.67 | 0.0336 |  |  |  |  |  |
| calreticulin | CALR_MOUSE | 1.5 | 5.33 | 0.0338 |  |  |  |  |  |
| ribosomal protein S5 | D3YYM6_MOUSE | 0 | 1.67 | 0.0353 |  |  |  |  |  |
| 60S ribosomal protein L7a | RL7A_MOUSE | 1.5 | 5.00 | 0.0387 |  |  |  |  |  |
| actin, alpha 1, skeletal muscle | ACTS_MOUSE | 0 | 1.33 | 0.0412 |  |  |  |  |  |
| Archain 1 | COPD_MOUSE | 0 | 1.33 | 0.0412 |  |  |  |  |  |
| catenin (cadherin associated protein), alpha 1 | CTNA1_MOUSE | 0 | 1.33 | 0.0412 |  |  |  |  |  |
| Type VI collagen A3 | E9Q8G0_MOUSE | 0 | 1.33 | 0.0412 |  |  |  |  |  |
| laminin, beta 2 | LAMB2_MOUSE | 0 | 1.33 | 0.0412 |  |  |  |  |  |
| guanine nucleotide binding protein (G protein), beta polypeptide 2 like 1 | GBLP_MOUSE | 0 | 1.67 | 0.0457 |  |  |  |  |  |
| protein phosphatase 2 (formerly 2A), regulatory subunit A (PR 65), alpha isoform | 2AAA_MOUSE | 0 | 2.00 | 0.0475 |  |  |  |  |  |
| annexin A11 | ANX11_MOUSE | 0 | 2.33 | 0.0480 |  |  |  |  |  |
| ATP citrate lyase | ACLY_MOUSE | 0 | 1.33 | 0.0494 |  |  |  |  |  |
| 60S ribosomal protein L19 | RL19_MOUSE | 0 | 1.33 | 0.0494 |  |  |  |  |  |
| ribophorin I | RPN1_MOUSE | 0 | 1.33 | 0.0494 |  |  |  |  |  |
| 40S ribosomal protein S23 | RS23_MOUSE | 0 | 1.33 | 0.0494 |  |  |  |  |  |

Proteins significantly increased in quantity in *Matn3* V194D cartilage extractions (buffer 3) relative to wild type (WT) control. Output from DAVID analysis is summarized by functionally related terms where (CC) denotes cellular compartment and (MF) denotes molecular function clusters; black boxes indicate membership of a grouping. Statistical significance was determined using the beta-binomial test and p<0.05 was considered significant.

| **Protein** | **Uniprot ID** | **WT spectra number** | **V194D spectra number** | **p value** | **Protein transport (BP)** |
| --- | --- | --- | --- | --- | --- |
| heterogeneous nuclear ribonucleoprotein U | HNRPU_MOUSE | 3.33 | 0 | 0.0006 |  |
| poly(rC) binding protein 1 | PCBP1_MOUSE | 2.33 | 0 | 0.0021 |  |
| proliferating cell nuclear antigen | PCNA_MOUSE | 2.33 | 0 | 0.0025 |  |
| coatomer protein delta-cop | COPD_MOUSE | 2 | 0 | 0.0039 |  |
| keratin 2 | K22E_MOUSE | 2 | 0 | 0.0039 |  |
| ceruloplasmin | CERU_MOUSE | 5 | 1 | 0.0121 |  |
| ELAV (embryonic lethal, abnormal vision, Drosophila)-like 1 (Hu antigen R) | ELAV1_MOUSE | 2 | 0 | 0.0172 |  |
| coatomer protein complex, subunit beta 2 (beta prime) | COPB2_MOUSE | 2.33 | 0 | 0.0173 |  |
| potassium channel tetramerisation domain containing 12 | KCD12_MOUSE | 1.33 | 0 | 0.0230 |  |
| spliceosome RNA helicase Ddx39b | DX39B_MOUSE | 2.67 | 0 | 0.0236 |  |
| GTP binding nuclear protein RAN | RAN_MOUSE | 1.33 | 0 | 0.0247 |  |
| ribosomal protein S3 | RS3_MOUSE | 1.33 | 0 | 0.0247 |  |
| spectrin alpha 2 | SPTA2_MOUSE | 1.33 | 0 | 0.0247 |  |
| procollagen-proline, 2-oxoglutarate 4-dioxygenase (proline 4-hydroxylase), alpha II polypeptide | P4HA2_MOUSE | 2.33 | 0 | 0.0255 |  |
| Rho GDP dissociation inhibitor (GDI) alpha | GDIR1_MOUSE | 3.33 | 0.67 | 0.0288 |  |
| thioredoxin domain containing 5 | TXND5_MOUSE | 2 | 0 | 0.0292 |  |
| ubiquitin-like modifier activating enzyme 1 | UBA1_MOUSE | 5 | 1.67 | 0.0293 |  |
| collagen, type XIV, alpha 1 | COEA1_MOUSE | 5.67 | 2 | 0.0309 |  |
| malate dehydrogenase 2, NAD (mitochondrial) | MDHM_MOUSE | 3.33 | 0.67 | 0.0312 |  |
| vinculin | VINC_MOUSE | 5 | 1.67 | 0.0313 |  |
| 40S ribosomal protein SA | RSSA_MOUSE | 1.33 | 0 | 0.0340 |  |
| lamin B1 | LMNB1_MOUSE | 1.67 | 0 | 0.0345 |  |
| proteasome (prosome, macropain) subunit, alpha type 1 | PSA1_MOUSE | 1.67 | 0 | 0.0345 |  |
| predicted gene 9234; peptidylprolyl isomerase A | PPIA_MOUSE | 4.67 | 1.67 | 0.0378 |  |

Proteins significantly decreased in quantity in *Matn3* V194D cartilage extractions (buffer 1) relative to wild type (WT) control. Output from DAVID analysis is summarized by functionally related terms where (BP) denotes biological process clusters; black boxes indicate membership of a grouping. Statistical significance was determined using the beta-binomial test and p<0.05 was considered significant.

| **Protein** | **Uniprot ID** | **WT spectra number** | **V194D spectra number** | **p value** | **Cell adhesion (BP)** | | **ECM (CC)** | | **Extracellular region (CC)** |
| --- | --- | --- | --- | --- | --- | --- | --- | --- | --- |
| matrilin 3 | MATN3_MOUSE | 4.33 | 0 | 0.0003 |  | |  | |  |
| histone H4 | H4_MOUSE | 1.67 | 0 | 0.0171 |  | |  | |  |
| keratin 2 | K22E_MOUSE | 1.67 | 0 | 0.0171 |  | |  | |  |
| epiphycan | EPYC_MOUSE | 3 | 0.67 | 0.0215 |  | |  | |  |
| integrin binding sialoprotein | SIAL_MOUSE | 1.67 | 0 | 0.0219 |  | |  | |  |
| decorin | PGS2_MOUSE | 4.67 | 0.67 | 0.0238 |  | |  | |  |
| plectin | PLEC_MOUSE | 1.67 | 0 | 0.0269 |  | |  | |  |
| secreted phosphoprotein 1 | OSTP_MOUSE | 3 | 0.67 | 0.0349 |  | |  | |  |
| collagen, type XIV, alpha 1 | COEA1_MOUSE | 4.33 | 1.67 | 0.0359 |  | |  | |  |
| collagen, type XII, alpha 1 | COCA1_MOUSE | 34.67 | 27.33 | 0.0421 |  | |  | |  |
| actinin alpha 4 | ACTN4_MOUSE | 4.67 | 1.67 | 0.0424 |  |  | |  | |

Proteins significantly decreased in quantity in *Matn3* V194D cartilage extractions (buffer 2) relative to wild type (WT) control. Output from DAVID analysis is summarized by functionally related terms of which (CC) denotes cellular compartment and (BP) denotes biological process clusters; black boxes indicate membership of a grouping. Statistical significance was determined using the beta-binomial test and p<0.05 was considered significant.

| **Protein** | **Uniprot ID** | **WT spectra number** | **V194D spectra number** | **p value** | **mitochondrion part (CC)** | **electron carrier activity (MF)** | **extracellular region (CC)** |
| --- | --- | --- | --- | --- | --- | --- | --- |
| desmoyokin | E9Q616_MOUSE | 7 | 0.00 | 0.0011 |  |  |  |
| dihydrolipoamide dehydrogenase | DLDH_MOUSE | 3 | 0.00 | 0.0031 |  |  |  |
| metastasis-associated gene family, member 2 | MTA2_MOUSE | 3 | 0.00 | 0.0031 |  |  |  |
| electron transferring flavoprotein, dehydrogenase | ETFD_MOUSE | 3.5 | 0.00 | 0.0031 |  |  |  |
| acyl-Coenzyme A dehydrogenase, medium chain | ACADM_MOUSE | 2.5 | 0.00 | 0.0047 |  |  |  |
| mitochondrial 10-formyltetrahydrofolate dehydrogenase | E9QLV8_MOUSE | 2.5 | 0.00 | 0.0047 |  |  |  |
| ribosomal protein L24 | RL24_MOUSE | 2.5 | 0.00 | 0.0047 |  |  |  |
| matrix metallopeptidase 2 | MMP2_MOUSE | 3 | 0.00 | 0.0058 |  |  |  |
| cysteine-rich secretory protein LCCL domain containing 1 | CRLD1_MOUSE | 2.5 | 0.00 | 0.0059 |  |  |  |
| protein S (alpha) | PROS_MOUSE | 2.5 | 0.00 | 0.0058 |  |  |  |
| ATP synthase, H+ transporting, mitochondrial F0 complex, subunit b, isoform 1 | AT5F1_MOUSE | 2 | 0.00 | 0.0084 |  |  |  |
| purine-nucleoside phosphorylase 1 | PNPH_MOUSE | 2 | 0.00 | 0.0084 |  |  |  |
| serine (or cysteine) peptidase inhibitor, clade A, member 1A | Q3KQQ4_MOUSE | 2 | 0.00 | 0.0084 |  |  |  |
| tenascin C | TENA_MOUSE | 21.5 | 10.67 | 0.0091 |  |  |  |
| spectrin alpha 2 | SPTA2_MOUSE | 6 | 1.67 | 0.0221 |  |  |  |
| actinin alpha 4 | ACTN4_MOUSE | 5.5 | 1.67 | 0.0279 |  |  |  |
| matrilin 3 | MATN3_MOUSE | 14.5 | 6.33 | 0.0312 |  |  |  |
| glutamate oxaloacetate transaminase 2, mitochondrial | AATM_MOUSE | 5 | 1.00 | 0.0348 |  |  |  |
| Chitinase 3-like protein 3 | CH3L3_MOUSE | 5 | 0.67 | 0.0360 |  |  |  |
| alpha-2-HS-glycoprotein | FETUA_MOUSE | 18.5 | 10.67 | 0.0367 |  |  |  |
| catalase | CATA_MOUSE | 4 | 1.00 | 0.0491 |  |  |  |

Proteins significantly decreased in quantity in *Matn3* V194D cartilage extractions (buffer 3) relative to wild type (WT) control. Output from DAVID analysis is summarized by functionally related terms of which (CC) denotes cellular compartment and (MF) denotes molecular function clusters; black boxes indicate membership of a grouping. Statistical significance was determined using the beta-binomial test and p<0.05 was considered significant.

| **Protein** | **Uniprot ID** | **WT spectra number** | **T585M spectra number** | **p value** | **Nuclear transport (BP)** | **Intracellular protein transport (BP)** | **Ribonucleotide binding (MF)** | **tRNA activation (BP)** | **Protein complex assembly (BP)** |
| --- | --- | --- | --- | --- | --- | --- | --- | --- | --- |
| chaperonin containing Tcp1, subunit 3 (gamma) | TCPG_MOUSE | 0 | 3 | 0.0010 |  |  |  |  |  |
| importin 5 | IPO5_MOUSE | 0 | 3 | 0.0016 |  |  |  |  |  |
| Bifunctional aminoacyl-tRNA synthetase | SYEP_MOUSE | 2 | 7.33 | 0.0073 |  |  |  |  |  |
| pyrophosphatase (inorganic) 1 | IPYR_MOUSE | 0 | 2 | 0.0164 |  |  |  |  |  |
| heterogeneous nuclear ribonucleoprotein A3 | ROA3_MOUSE | 0 | 1.67 | 0.0190 |  |  |  |  |  |
| topoisomerase (DNA) II alpha | TOP2A_MOUSE | 0 | 2 | 0.0203 |  |  |  |  |  |
| coronin 7 | CORO7_MOUSE | 0 | 1.67 | 0.0222 |  |  |  |  |  |
| karyopherin (importin) beta 1 | IMB1_MOUSE | 0.67 | 3.33 | 0.0227 |  |  |  |  |  |
| ribonucleotide reductase M1 | RIR1_MOUSE | 0 | 1.67 | 0.0240 |  |  |  |  |  |
| lysyl-tRNA synthetase | SYK_MOUSE | 0 | 1.67 | 0.0240 |  |  |  |  |  |
| fatty acid synthase | FAS_MOUSE | 2 | 7.33 | 0.0242 |  |  |  |  |  |
| ATP-binding cassette, sub-family E (OABP), member 1 | ABCE1_MOUSE | 0 | 1.33 | 0.0246 |  |  |  |  |  |
| CD109 antigen | CD109_MOUSE | 0 | 1.33 | 0.0246 |  |  |  |  |  |
| DEAD (Asp-Glu-Ala-Asp) box polypeptide 17 | DDX17_MOUSE | 0 | 1.33 | 0.0246 |  |  |  |  |  |
| predicted gene 13886; TAR DNA binding protein | TADBP_MOUSE | 0 | 1.33 | 0.0246 |  |  |  |  |  |
| cullin associated and neddylation disassociated 1 | CAND1_MOUSE | 1.33 | 5 | 0.0259 |  |  |  |  |  |
| spectrin beta 2 | SPTB2_MOUSE | 1 | 5 | 0.0270 |  |  |  |  |  |
| oxoglutarate dehydrogenase (lipoamide) | ODO1_MOUSE | 0 | 2 | 0.0284 |  |  |  |  |  |
| hexokinase 1 | HXK1_MOUSE | 0 | 1.33 | 0.0287 |  |  |  |  |  |
| chromosome segregation 1-like (S. cerevisiae) | XPO2_MOUSE | 0 | 1.33 | 0.0287 |  |  |  |  |  |
| coatomer protein complex subunit alpha | COPA_MOUSE | 3.33 | 8.67 | 0.0288 |  |  |  |  |  |
| aldehyde dehydrogenase 9, subfamily A1 | AL9A1_MOUSE | 0 | 1.33 | 0.0379 |  |  |  |  |  |
| G protein pathway suppressor 1 | CSN1_MOUSE | 0 | 1.33 | 0.0379 |  |  |  |  |  |
| eukaryotic translation initiation factor 3, subunit C | EIF3C_MOUSE | 0 | 1.33 | 0.0379 |  |  |  |  |  |
| alanyl-tRNA synthetase | SYAC_MOUSE | 0.67 | 3 | 0.0386 |  |  |  |  |  |
| ribosome binding protein 1 | RRBP1_MOUSE | 2 | 5.67 | 0.0467 |  |  |  |  |  |

**Table S2.**

Proteins significantly increased in quantity in *Comp* T585M cartilage extractions (buffer 1) relative to wild type (WT) control. Output from DAVID analysis is summarized by functionally related terms where (MF) denotes molecular function and (BP) denotes biological process clusters; black boxes indicate membership of a grouping. Statistical significance was determined using the beta-binomial test and p<0.05 was considered significant.

| **Protein** | **Uniprot ID** | **WT spectra number** | **T585M spectra number** | **p value** | **Actin cytoskeleton (CC)** |
| --- | --- | --- | --- | --- | --- |
| vimentin | VIME_MOUSE | 0 | 3 | 0.0015 |  |
| high mobility group protein 1 | HMGB1_MOUSE | 0 | 3 | 0.0016 |  |
| ribosomal protein S3 | RS3_MOUSE | 0 | 2.67 | 0.0033 |  |
| protein disulfide isomerase associated 6 | PDIA6_MOUSE | 0 | 3 | 0.0034 |  |
| succinate dehydrogenase complex, subunit A, flavoprotein (Fp) | DHSA_MOUSE | 0 | 2.33 | 0.0040 |  |
| acidic (leucine-rich) nuclear phosphoprotein 32 family, member B | AN32B_MOUSE | 0 | 2.33 | 0.0056 |  |
| spectrin alpha 2 | SPTA2_MOUSE | 0 | 2 | 0.0230 |  |
| talin 1 | TLN1_MOUSE | 1 | 5.67 | 0.0246 |  |
| myosin, heavy polypeptide 4, skeletal muscle | MYH4_MOUSE | 0 | 7 | 0.0300 |  |
| synaptotagmin binding, cytoplasmic RNA interacting protein | HNRPQ_MOUSE | 0 | 1.33 | 0.0318 |  |
| liver glycogen phosphorylase | PYGL_MOUSE | 0 | 1.33 | 0.0318 |  |
| phosphatase 2A inhibitor | SET_MOUSE | 0 | 1.33 | 0.0318 |  |
| periostin, osteoblast specific factor | POSTN_MOUSE | 0 | 1.33 | 0.0404 |  |
| ribonuclease, RNase A family 4 | RNAS4_MOUSE | 0 | 1.67 | 0.0410 |  |

Proteins significantly increased in quantity in *Comp* T585M cartilage extractions (buffer 2) relative to wild type (WT) control. Output from DAVID analysis is summarized by functionally related terms where (CC) denotes cellular compartment cluster; black boxes indicate membership of a grouping. Statistical significance was determined using the beta-binomial test and p<0.05 was considered significant.

| **Protein** | **Uniprot ID** | **WT spectra number** | **T585M spectra number** | **p value** | **ECM (CC)** |
| --- | --- | --- | --- | --- | --- |
| hexosaminidase B | HEXB_MOUSE | 0 | 3.67 | 0.0019 |  |
| tubulin, beta 2A | TBB2A_MOUSE | 0 | 3.67 | 0.0027 |  |
| ribosomal protein S9 | RS9_MOUSE | 0 | 3.00 | 0.0036 |  |
| poly(rC) binding protein 1 | PCBP1_MOUSE | 0 | 2.67 | 0.0048 |  |
| laminin, beta 2 | LAMB2_MOUSE | 0 | 2.67 | 0.0048 |  |
| proteasome (prosome, macropain) subunit, alpha type 6 | PSA6_MOUSE | 0 | 2.00 | 0.0132 |  |
| ribosomal protein L15 | RL15_MOUSE | 0 | 2.00 | 0.0132 |  |
| actin, alpha 1, skeletal muscle | ACTS_MOUSE | 0 | 2.00 | 0.0282 |  |
| matrix metallopeptidase 9 | MMP9_MOUSE | 0 | 2.33 | 0.0300 |  |
| collagen, type XIV, alpha 1 | COEA1_MOUSE | 0 | 3.00 | 0.0346 |  |
| protease (prosome, macropain) 26S subunit, ATPase 5 | PRS8_MOUSE | 0 | 1.67 | 0.0350 |  |
| transketolase | TKT_MOUSE | 0 | 1.67 | 0.0350 |  |
| Bifunctional aminoacyl-tRNA synthetase | SYEP_MOUSE | 0 | 1.67 | 0.0354 |  |
| periostin, osteoblast specific factor | POSTN_MOUSE | 0 | 3.67 | 0.0381 |  |
| collagen, type IX, alpha 1 | Q8BSQ4_MOUSE | 2 | 7.33 | 0.0400 |  |
| collagen, type VI, alpha 6 | CO6A6_MOUSE | 0 | 2.00 | 0.0418 |  |
| guanine nucleotide binding protein (G protein), beta polypeptide 2 like 1 | GBLP_MOUSE | 0 | 1.33 | 0.0438 |  |
| integrin binding sialoprotein | SIAL_MOUSE | 0 | 1.33 | 0.0438 |  |
| TNF receptor-associated protein 1 | TRAP1_MOUSE | 0 | 1.33 | 0.0438 |  |

Proteins significantly increased in quantity in *Comp* T585M cartilage extractions (buffer 3), relative to wild type (WT) control. Output from DAVID analysis is summarized by functionally related terms where (CC) denotes cellular compartment clusters; black boxes indicate membership of a grouping. Statistical significance was determined using the beta-binomial test and p<0.05 was considered significant.

| **Protein** | **Uniprot ID** | **WT spectra number** | **T585M spectra number** | **p value** | **ECM (CC)** |
| --- | --- | --- | --- | --- | --- |
| coagulation factor XIII, A1 subunit | F13A_MOUSE | 2.33 | 0 | 0.0018 |  |
| collagen, type XI, alpha 1 | COBA1_MOUSE | 5.33 | 0.67 | 0.0124 |  |
| collagen, type IX, alpha 1 | CO9A1_MOUSE | 2 | 0 | 0.0160 |  |
| ELAV (embryonic lethal, abnormal vision, Drosophila)-like 1 (Hu antigen R) | ELAV1_MOUSE | 2 | 0 | 0.0160 |  |
| Murinoglobulin 1 | MUG1_MOUSE | 1.67 | 0 | 0.0191 |  |
| secreted acidic cysteine rich glycoprotein | SPRC_MOUSE | 1.67 | 0 | 0.0209 |  |
| ribonuclease, RNase A family 4 | RNAS4_MOUSE | 2 | 0 | 0.0219 |  |
| GTP-binding nuclear protein Ran | RAN_MOUSE | 1.33 | 0 | 0.0224 |  |
| ribosomal protein S3 | RS3_MOUSE | 1.33 | 0 | 0.0224 |  |
| creatine kinase, brain | KCRB_MOUSE | 3 | 0.67 | 0.0234 |  |
| malate dehydrogenase 2, NAD (mitochondrial) | MDHM_MOUSE | 3.33 | 0.67 | 0.0282 |  |
| Aspartate aminotransferase, cytoplasmic | AATC_MOUSE | 1.33 | 0 | 0.0313 |  |
| catalase | CATA_MOUSE | 1.33 | 0 | 0.0313 |  |
| cofilin 1, non-muscle | COF1_MOUSE | 1.33 | 0 | 0.0313 |  |
| milk fat globule-EGF factor 8 protein | MFGM_MOUSE | 1.33 | 0 | 0.0313 |  |
| proteasome (prosome, macropain) subunit, alpha type 1 | PSA1_MOUSE | 1.67 | 0 | 0.0323 |  |
| Rho GDP dissociation inhibitor (GDI) alpha | GDIR1_MOUSE | 3.33 | 0.67 | 0.0345 |  |

Proteins significantly decreased in quantity in *Comp* T585M cartilage extractions (buffer 1) relative to wild type (WT) control. Output from DAVID analysis is summarized by functionally related terms where (CC) denotes cellular compartment clusters; black boxes indicate membership of a grouping. Statistical significance was determined using the beta-binomial test and p<0.05 was considered significant.

| **Protein** | **Uniprot ID** | **WT spectra number** | **T585M spectra number** | **p value** | **ECM(CC)** |
| --- | --- | --- | --- | --- | --- |
| sushi-repeat-containing protein | SRPX_MOUSE | 2 | 0 | 0.0022 |  |
| AE binding protein 1 | AEBP1_MOUSE | 2.33 | 0 | 0.0149 |  |
| 60S ribosomal protein L12 | RL12_MOUSE | 1.67 | 0 | 0.0167 |  |
| Glyceraldehyde-3-phosphate dehydrogenase (GAPDH) | G3P_MOUSE | 1.33 | 0 | 0.0173 |  |
| hyaluronic acid binding protein 2 | HABP2_MOUSE | 1.33 | 0 | 0.0211 |  |
| hemoglobin subunit beta-1 | HBB1_MOUSE | 2.67 | 0.67 | 0.0217 |  |
| histone cluster 1, H1a | H11_MOUSE | 1.67 | 0 | 0.0247 |  |
| fibronectin 1 | FINC_MOUSE | 12.67 | 9 | 0.0248 |  |
| vitronectin | VTNC_MOUSE | 2.67 | 0.67 | 0.0261 |  |
| matrix metallopeptidase 13 | MMP13_MOUSE | 4.33 | 2 | 0.0387 |  |
| collagen, type XII, alpha 1 | COCA1_MOUSE | 34.67 | 30.67 | 0.0393 |  |

Proteins significantly decreased in quantity in *Comp* T585M cartilage extractions (buffer 2) relative to wild type (WT) control. Output from DAVID analysis is summarized by functionally related terms where (CC) denotes cellular compartment clusters; black boxes indicate membership of a grouping. Statistical significance was determined using the beta-binomial test and p<0.05 was considered significant.

| **Protein** | **Uniprot ID** | **WT spectra number** | **T585M spectra number** | **p value** | **Ribosome (CC)** |
| --- | --- | --- | --- | --- | --- |
| alpha-2-HS-glycoprotein | FETUA_MOUSE | 18.5 | 10.67 | 0.0285 |  |
| protein kinase C substrate 80K-H | GLU2B_MOUSE | 2 | 0.00 | 0.0078 |  |
| metastasis-associated gene family, member 2 | MTA2_MOUSE | 3 | 0.00 | 0.0029 |  |
| myosin IC | MYO1C_MOUSE | 2.5 | 0.00 | 0.0044 |  |
| protein S (alpha) | PROS_MOUSE | 2.5 | 0.00 | 0.0055 |  |
| ribosomal protein L24 | RL24_MOUSE | 2.5 | 0.00 | 0.0044 |  |
| 60S ribosomal protein L6 | RL6_MOUSE | 5.5 | 1.33 | 0.0348 |  |
| 40S ribosomal protein S8 | RS8_MOUSE | 3 | 0.67 | 0.0495 |  |

Proteins significantly decreased in quantity in *Comp* T585M cartilage extractions (buffer 3) relative to wild type (WT) control. Output from DAVID analysis is summarized by functionally related terms where (CC) denotes cellular compartment clusters; black boxes indicate membership of a grouping. Statistical significance was determined using the beta-binomial test and p<0.05 was considered significant.

**Table S3**

| **Protein** | **Uniprot ID** | **WT spectra number** | **D469del spectra number** | **p value** |
| --- | --- | --- | --- | --- |
| immunoglobulin heavy constant gamma-1 | IGH1M_MOUSE | 0 | 2.67 | 0.0019 |
| chitinase 3-like 3 | CH3L3_MOUSE | 0.67 | 4 | 0.0078 |
| DNA replication licensing factor MCM2 | MCM2_MOUSE | 0.67 | 3.33 | 0.0119 |
| threonyl-tRNA synthetase | SYTC_MOUSE | 0 | 2 | 0.0173 |
| 3'-phosphoadenosine 5'-phosphosulfate synthase 2 | PAPS2_MOUSE | 0 | 1.67 | 0.0176 |
| muscle glycogen phosphorylase | PYGM_MOUSE | 0 | 1.67 | 0.0176 |
| ribonuclease/angiogenin inhibitor 1 | RINI_MOUSE | 0 | 2 | 0.0197 |
| pre-mRNA processing factor 18 homolog | PRP18_MOUSE | 0 | 1.33 | 0.0205 |
| Glucosidase 2 subunit beta | GLU2B_MOUSE | 1.33 | 4.33 | 0.0206 |
| splicing factor 3b, subunit 3 | SF3B3_MOUSE | 0 | 1.67 | 0.0216 |
| pyrophosphatase (inorganic) 1 | IPYR_MOUSE | 0 | 2.67 | 0.0258 |
| coronin 7 | CORO7_MOUSE | 0 | 1.33 | 0.0301 |
| malate dehydrogenase 2, NAD (mitochondrial) | MDHM_MOUSE | 3.33 | 0.67 | 0.0397 |

Proteins significantly increased in quantity in *Comp* D469del cartilage extractions (buffer 1) relative to wild type (WT) control. No clusters of terms were detected using DAVID. Statistical significance was determined using the beta-binomial test p<0.05 was considered significant.

| **Protein** | **Uniprot ID** | **WT spectra number** | **D469del spectra number** | **p value** | **ER (CC)** | **ECM (CC)** |
| --- | --- | --- | --- | --- | --- | --- |
| leprecan 1 | P3H1_MOUSE | 0 | 2.67 | 0.0021 |  |  |
| vimentin | VIME_MOUSE | 0 | 2.33 | 0.0028 |  |  |
| high mobility group box 2 | HMGB2_MOUSE | 0 | 2.33 | 0.0031 |  |  |
| ribosomal protein S3 | RS3_MOUSE | 0 | 2.33 | 0.0031 |  |  |
| annexin A3 | ANXA3_MOUSE | 0 | 2 | 0.0052 |  |  |
| collagen, type I, alpha 1 | CO1A1_MOUSE | 0 | 2 | 0.0059 |  |  |
| cartilage associated protein | CRTAP_MOUSE | 0 | 1.67 | 0.0209 |  |  |
| lymphocyte cytosolic protein 1 | PLSL_MOUSE | 0 | 1.67 | 0.0209 |  |  |
| heat shock protein 90 | HS90A_MOUSE | 0 | 2.67 | 0.0209 |  |  |
| acidic ribosomal phosphoprotein P0 | RLA0_MOUSE | 0 | 1.33 | 0.0248 |  |  |
| thrombospondin 4 | TSP4_MOUSE | 0 | 1.33 | 0.0343 |  |  |
| protein disulfide isomerase associated 6 | PDIA6_MOUSE | 0 | 2.33 | 0.0372 |  |  |
| myosin, heavy polypeptide 4, skeletal muscle | MYH4_MOUSE | 0 | 2 | 0.0396 |  |  |
| calreticulin | CALR_MOUSE | 1 | 4 | 0.0453 |  |  |

Proteins significantly increased in quantity in *Comp* D469del cartilage extractions (buffer 2) relative to wide type (WT) control. Output from DAVID analysis is summarized by functionally related terms where (CC) denotes cellular compartment clusters; black boxes indicate membership of a grouping. Statistical significance was determined using the beta-binomial test and p<0.05 was considered significant.

| **Protein** | **Uniprot ID** | **WT spectra number** | **D469del spectra number** | **p value** | **Membranebound vesicle (CC)** |
| --- | --- | --- | --- | --- | --- |
| ribosomal protein S9 | RS9_MOUSE | 0.00 | 3.67 | 0.0012 |  |
| 40S ribosomal protein | RS27_MOUSE | 0.00 | 2.67 | 0.0035 |  |
| poly(rC) binding protein 1 | PCBP1_MOUSE | 0.00 | 2.33 | 0.0055 |  |
| transketolase | TKT_MOUSE | 0.00 | 2.00 | 0.0083 |  |
| cathepsin G | CATG_MOUSE | 0.00 | 1.67 | 0.0236 |  |
| leucine rich repeat containing 59 | LRC59_MOUSE | 0.00 | 1.67 | 0.0236 |  |
| tyrosine 3-monooxygenase/tryptophan 5-monooxygenase activation protein | 1433Z_MOUSE | 0.00 | 1.67 | 0.0322 |  |
| hexosaminidase B | HEXB_MOUSE | 0.00 | 1.67 | 0.0322 |  |
| citrate synthase | CISY_MOUSE | 0.00 | 2.00 | 0.0329 |  |
| fibulin 7 | FBLN7_MOUSE | 0.00 | 1.33 | 0.0331 |  |
| phosphoribosylaminoimidazole carboxylase | PUR6_MOUSE | 0.00 | 1.33 | 0.0331 |  |
| serine (or cysteine) peptidase inhibitor, clade H, member 1 | SERPH_MOUSE | 21.00 | 26.67 | 0.0369 |  |
| eukaryotic translation initiation factor 4A1 | IF4A1_MOUSE | 0.00 | 2.33 | 0.0386 |  |
| annexin A1 | ANXA1_MOUSE | 5.00 | 8.67 | 0.0492 |  |

Proteins significantly increased in quantity in *Comp* D469del cartilage extractions (buffer 3) relative to wild type (WT) control. Output from DAVID analysis is summarized by functionally related terms where (CC) denotes cellular compartment clusters; black boxes indicate membership of a grouping. Statistical significance was determined using the beta-binomial test and p<0.05 was considered significant.

| **Protein** | **Uniprot ID** | **WT spectra number** | **D469del spectra number** | **p value** | **Organelle lumen (CC)** |
| --- | --- | --- | --- | --- | --- |
| Rho GDP dissociation inhibitor (GDI) alpha | GDIR1_MOUSE | 3.33 | 0 | 0.00213 |  |
| proliferating cell nuclear antigen | PCNA_MOUSE | 2.33 | 0 | 0.00319 |  |
| collagen, type XIV, alpha 1 | COEA1_MOUSE | 5.67 | 0.67 | 0.008 |  |
| adseverin | ADSV_MOUSE | 2 | 0 | 0.01918 |  |
| collagen, type IX, alpha 1 | CO9A1_MOUSE | 2 | 0 | 0.01918 |  |
| ELAV (embryonic lethal, abnormal vision, Drosophila)-like 1 (Hu antigen R) | ELAV1_MOUSE | 2 | 0 | 0.01918 |  |
| spliceosome RNA helicase Ddx39b | DX39B_MOUSE | 2.67 | 0 | 0.02573 |  |
| ribonuclease, RNase A family 4 | RNAS4_MOUSE | 2 | 0 | 0.02608 |  |
| keratin 17 | K1C17_MOUSE | 2.33 | 0 | 0.02836 |  |
| thioredoxin domain containing 5 | TXND5_MOUSE | 2 | 0 | 0.03225 |  |
| serine (or cysteine) peptidase inhibitor, clade H, member 1 | SERPH_MOUSE | 6.33 | 2 | 0.03439 |  |
| proteasome (prosome, macropain) subunit, alpha type 1 | PSA1_MOUSE | 1.67 | 0 | 0.03835 |  |
| Aspartate aminotransferase, cytoplasmic | AATC_MOUSE | 1.33 | 0 | 0.03847 |  |
| catalase | CATA_MOUSE | 1.33 | 0 | 0.03847 |  |
| malate dehydrogenase 2, NAD (mitochondrial) | MDHM_MOUSE | 3.33 | 0.67 | 0.03967 |  |

Proteins significantly decreased in quantity in *Comp* D469del cartilage extractions (buffer 1) relative to wild type (WT) control. Output from DAVID analysis is summarized by functionally related terms where (CC) denotes cellular compartment clusters; black boxes indicate membership of a grouping. Statistical significance was determined using the beta-binomial test and p<0.05 was considered significant.

| **Protein** | **Uniprot ID** | **WT spectra number** | **D469del spectra number** | **p value** | **Extracellular region (CC)** |
| --- | --- | --- | --- | --- | --- |
| thrombospondin 3 | TSP3_MOUSE | 2.67 | 0 | 0.0014 |  |
| procollagen C-endopeptidase enhancer 2 | SRPX_MOUSE | 2 | 0 | 0.0030 |  |
| sushi-repeat-containing protein | AEBP1_MOUSE | 2.33 | 0 | 0.0165 |  |
| integrin binding sialoprotein | PROS_MOUSE | 1.67 | 0 | 0.0176 |  |
| epiphycan | EPYC_MOUSE | 3 | 0.67 | 0.0191 |  |
| protein S (alpha) | G3P_MOUSE | 1.33 | 0 | 0.0201 |  |
| collagen, type XIV, alpha 1 | COEA1_MOUSE | 4.33 | 1.33 | 0.0210 |  |
| glyceraldehyde-3-phosphate dehydrogenase | SIAL_MOUSE | 1.67 | 0 | 0.0225 |  |
| AE binding protein 1 | PCOC2_MOUSE | 2.33 | 0 | 0.0286 |  |

Proteins significantly decreased in quantity in *Comp* D469del cartilage extractions (buffer 2) relative to wild type (WT) control. Output from DAVID analysis is summarized by functionally related terms where (CC) denotes cellular compartment clusters; black boxes indicate membership of a grouping. Statistical significance was determined using the beta-binomial test and p<0.05 was considered significant.

| **Protein** | **Uniprot ID** | **WT spectra number** | **D469del spectra number** | **p value** | **ECM (CC)** |
| --- | --- | --- | --- | --- | --- |
| spectrin alpha 2 | SPTA2_MOUSE | 6.00 | 0.00 | 0.0008 |  |
| electron transferring flavoprotein, dehydrogenase | ETFD_MOUSE | 3.50 | 0.00 | 0.0036 |  |
| acyl-Coenzyme A dehydrogenase, medium chain | ACADM_MOUSE | 2.50 | 0.00 | 0.0057 |  |
| procollagen C-endopeptidase enhancer 2 | PCOC2_MOUSE | 2.50 | 0.00 | 0.0057 |  |
| programmed cell death 6 interacting protein | PDC6I_MOUSE | 2.50 | 0.00 | 0.0057 |  |
| proteasome (prosome, macropain) 26S subunit, non-ATPase, 11 | PSD11_MOUSE | 2.50 | 0.00 | 0.0057 |  |
| ribosomal protein L24 | RL24_MOUSE | 2.50 | 0.00 | 0.0057 |  |
| cysteine-rich secretory protein LCCL domain containing 1 | CRLD1_MOUSE | 2.50 | 0.00 | 0.0069 |  |
| protein S (alpha) | PROS_MOUSE | 2.50 | 0.00 | 0.0069 |  |
| DNA segment, Chr 1, Pasteur Institute 1 | DDX3L_MOUSE | 2.00 | 0.00 | 0.0107 |  |
| protein kinase C substrate 80K-H | GLU2B_MOUSE | 2.00 | 0.00 | 0.0107 |  |
| proteasome (prosome, macropain) subunit, beta type 5 | PSB5_MOUSE | 2.00 | 0.00 | 0.0107 |  |
| tenascin X | O35452_MOUSE | 10.00 | 1.00 | 0.0135 |  |
| tenascin C | TENA_MOUSE | 21.50 | 7.67 | 0.0199 |  |
| transforming growth factor, beta induced protein ig-h3 | BGH3_MOUSE | 4.00 | 0.67 | 0.0213 |  |
| actinin alpha 4 | ACTN4_MOUSE | 5.50 | 1.33 | 0.0332 |  |
| 60S ribosomal protein L6 | RL6_MOUSE | 5.50 | 1.00 | 0.0334 |  |
| collagen, type VI, alpha 1 | CO6A1_MOUSE | 14.50 | 6.67 | 0.0335 |  |
| Nidogen 1 | NID1_MOUSE | 3.50 | 0.67 | 0.0386 |  |
| desmoyokin | E9Q616_MOUSE | 7.00 | 1.67 | 0.0438 |  |

Proteins significantly decreased in quantity in *Comp* D469del cartilage extractions (buffer 3) relative to wild type (WT) control. Output from DAVID analysis is summarized by functionally related terms where (CC) denotes cellular compartment clusters; black boxes indicate membership of a grouping. Statistical significance was determined using the beta-binomial test and p<0.05 was considered significant.
